# Diarylethene-Based Ionic Liquids: Synthesis and Photo-Driven Solution Properties

**DOI:** 10.3390/ijms24043533

**Published:** 2023-02-09

**Authors:** Mário R. C. Soromenho, Carlos A. M. Afonso, José M. S. S. Esperança

**Affiliations:** 1 LAQV-REQUIMTE, Departamento de Química, Faculdade de Ciências e Tecnologia, Universidade NOVA de Lisboa, 2829-516 Caparica, Portugal; 2 Research Institute for Medicine (iMed.ULisboa), Faculty of Pharmacy, Universidade de Lisboa, 1649-003 Lisboa, Portugal

**Keywords:** diarylethene, ionic liquids, photo-driven properties, photochromic salts

## Abstract

In this work, the design and synthesis of a series of photochromic gemini diarylethene-based ionic liquids (GDILs) with different cationic motifs is reported. Several synthetic pathways were optimized for the formation of cationic GDILs with chloride as the counterion. The different cationic motifs were achieved through the *N*-alkylation of the photochromic organic core unit with different tertiary amines, including different aromatic amines such as imidazole derivatives and pyridinium, and other non-aromatic amines. These novel salts present surprising water solubility with unexplored photochromic features that broaden their known applications. The covalent attachment of the different side groups dictates their water solubility and differences upon photocyclization. The physicochemical properties of GDILs in aqueous and in imidazolium-based ionic liquid (IL) solutions were investigated. Upon irradiation with ultraviolet (UV) light, we have observed changes in the physico-chemical properties of distinct solutions containing these GDILs, at very low concentrations. More specifically, in aqueous solution, the overall conductivity increased with the time of UV photoirradiation. In contrast, in IL solution, these photoinducible changes are dependent on the type of ionic liquid used. These compounds can improve non-ionic and ionic liquids’ solutions since we can change their properties, such as conductivity, viscosity or ionicity, only by UV photoirradiation. The electronic and conformational changes associated with these innovative stimuli GDILs may open new opportunities for their use as photoswitchable materials.

## 1. Introduction

Currently, the interest in light-driven reversible intramolecular switches such as photochromic materials increased exponentially [1]. An ideal photochromic compound should have high efficiency, but thermal stability, fatigue resistance and quantum yield also play an important role in the field of applications [1,2]. The chemical structure and physicochemical properties of photochromic materials can be fine-tuned by the proper choice of their photochromic cores and chemical derivatizations [1]. Diarylethene cores represent a very promising class of photochromic centres due to their high thermal stability and fatigue resistance, [3,4,5] undergoing a reversible structural change between an open (colourless) and a closed (coloured) form by light induction [4,6,7,8,9,10]. These structural and electronic transformations create important changes in the physical and chemical properties of the photochromic media, such as geometry, [11,12] conductivity, [13] refraction, [14,15,16,17,18,19] and solubility [20].

**Scheme 1:**
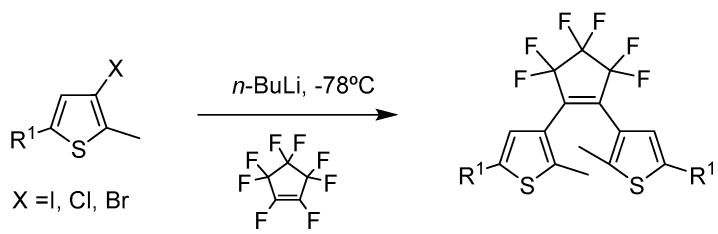
Strategic approach for the synthesis of diarylethene derivatives.

Nowadays, sulfur heterocyclic compounds are commonly used as precursors in organic synthesis. Thiophene is one of the most studied five membered ring heterocyclic compounds due to its abundancy and versatility. The continuous development of thiophenic compounds is mainly due to the capacity to fine tune their structural functionalization [21,22]. This ability is the driving force for tailoring the photophysical properties of thiophene for a wide range of applications in materials science. [23,24,25,26,27,28,29] From a literature perspective, the general synthetic procedure for diarylethene-based molecules are generally reported as shown in Scheme 1 [21].

Generally, diarylethene derivatives are water insoluble, but the solubility in water can increase by linking the photochromic core unit to highly hydrophilic moieties such as carbohydrates [30] or water-soluble polymeric structures [31].

Ionic liquids (ILs) are a well-known class of substances composed entirely of ions, with low melting temperatures and unique properties [32,33,34,35]. The possibility of designing their structure to tune the properties has driven ionic liquids research to a large number of potential applications with some already at industrial scale [36,37,38]. The incorporation of a diarylethene centre as a substituent unit in the ionic liquid cation leads to a bifunctional material [39,40]. This symbiosis can enhance the specific properties of the material, with a relevant effect, e.g., on the melting temperature, thermal stability, viscosity, conductivity, polarity, or even in its miscibility with common solvents, namely water. This can create a platform of new reversible water soluble light responsive materials with promising applications [39,40,41,42].

**Scheme 2:**
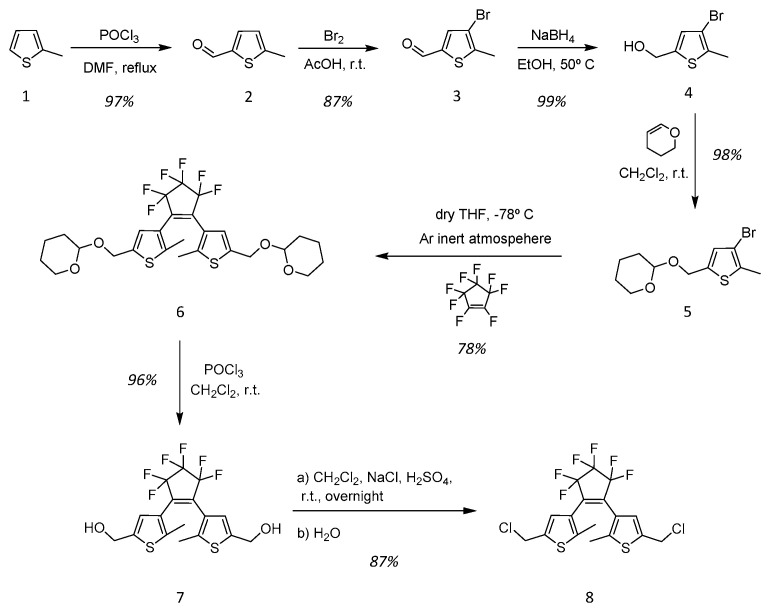
Optimized synthetic pathway for diarylethene alkylating agent.

A new methodology for the preparation of diarylethene (DAE) derivatives is described, using thiophenes as the heteroaromatic rings, differing on the R^1^ substituents for different property behaviours and choosing fluorinated cyclopentenes as bridging motifs. These will be important building blocks for the synthesis of new diarylethene-based molecules, which can lead to the creation of novel diarylethene-based ionic liquids with distinctive and exclusive properties. The following strategy for the different thiophenic derivatives was implemented, as shown in Scheme 2.

**Scheme 3:**
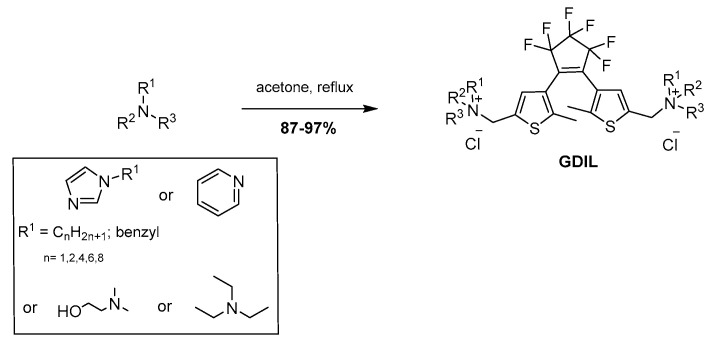
General synthetic procedure of gemini diarylethene based ionic liquids (GDILs).

The GDILs used in this work were prepared from direct N-alkylation of a diarylethene molecule that bears a chlorinated moiety at the terminus with different tertiary amines (cyclic and non-cyclic) to give the respective cationic species (Scheme 3) in good yields.

**Figure 1:**
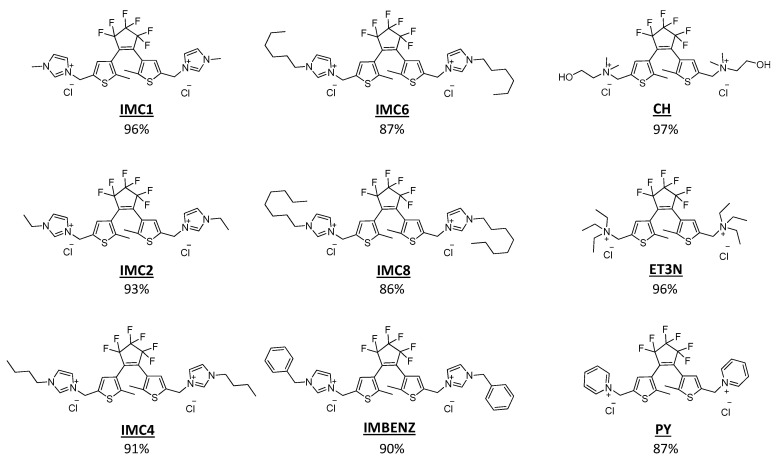
Chemical structure of synthesized GDILs.

In this work, we synthesised water soluble gemini diarylethene-based ionic liquids (GDILs) using imidazolium, ammonium or pyridinium as cations with chloride as the counterion (Figure 1) and studied how its photoconversion with UV irradiation can influence the properties of common ionic liquids. Previous studies showed that irradiating neutral photochromic molecules in ionic liquid media can enhance their photoconversion [43]. Coleman and coworkers showed that spiropyrans salts can influence the properties in ionic liquid solutions even at 1 mol% concentration [44]. Here, we report the potential application of distinct GDILs, to control, by UV irradiation, the properties of ionic liquid solutions, at very low concentrations. Interestingly, the changes in distinct photoinducible solution properties depend on the dicationic moiety present.

## 2. Results and Discussion

### 2.1. Influence of GDILs Photoconversion in the Ionic Conductivity in Solution

In this work, we have synthesized several water-soluble GDILs (Figure 1). All GDILs have the same anion, chloride, and the same diarylethene core attached to the cation, only differing on their ionic adjacent moiety. Since these compounds are solid, we studied their properties in solution, at a given concentration, for a chosen solvent system. These concentrations are determined by the lowest GDIL solubility, to be able to compare all GDILs in solution. For this study, we have chosen water and different ionic liquids with distinct characteristics, as solvent media, to determine their photoinducible effect in solution. The ionic liquids used include a hydrophilic ionic liquid, 1-ethyl-3-methylimidazolium thiocyanate, [C_2_mim][SCN], known to be one of the more conductive and less viscous ionic liquids in literature and a hydrophobic ionic liquid, 1-ethyl-3-methylimidazolium bistrifluoromethanesulfonylimide, [C_2_mim][NTf_2_], which is known to be one of the more stable hydrophobic ionic liquids. Since very few studies in literature describe the photocyclization effect in aqueous solutions, [30,31] we decided to start evaluating this effect for this solvent system. We determined the maximum solubility of the open form GDILs in water, to show the dicationic moiety influence in this property. Due to the high hygroscopicity of these GDILs, the methodology chosen was to prepare a solution with a precise amount of GDIL above the solubility limit (solid GDIL suspended in the solution). Then, a stepwise addition of water to the solution was performed to achieve a final homogeneous mixture. The results are presented in Table 1. As expected, the type of cationic moiety has a high effect on the solubility of these diarylethene salts. These results represent a key step onto the development of tunable diarylethene salts with distinct cationic motifs to achieve higher solubility in water. In water, PY presents the lowest solubility, with 6.73 × 10^−4^ mol%.

**Scheme 4:**
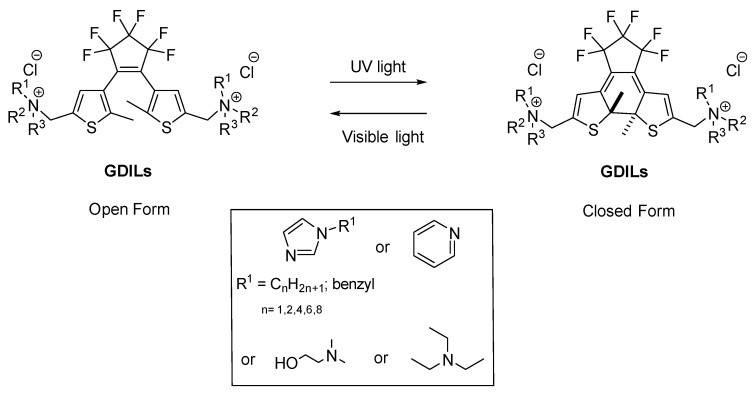
General structures of gemini diarylethene based ionic liquids (GDILs) in the open and closed forms.

**Figure 2:**
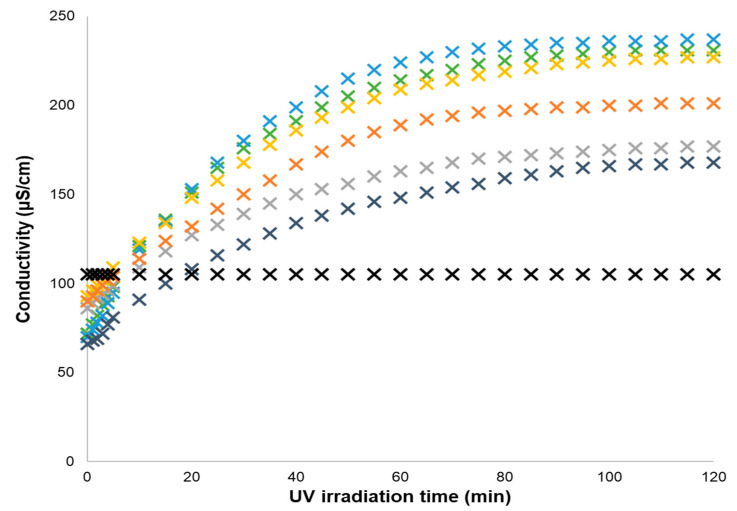
Conductivity data of 6.73 × 10^−4^ mol% GDIL IMCn series and of 1.35 × 10^−3^ mol% KCl aqueous solutions upon UV irradiation time at 25 °C (**X** black cross—KCl, **X** green cross—IMC1, **X** blue cross—IMC2, **X** gold cross—IMC4, **X** gray cross –IMC6, **X** orange cross—IMC8, **X** blue-gray cross—IMBENZ).

**Figure 3:**
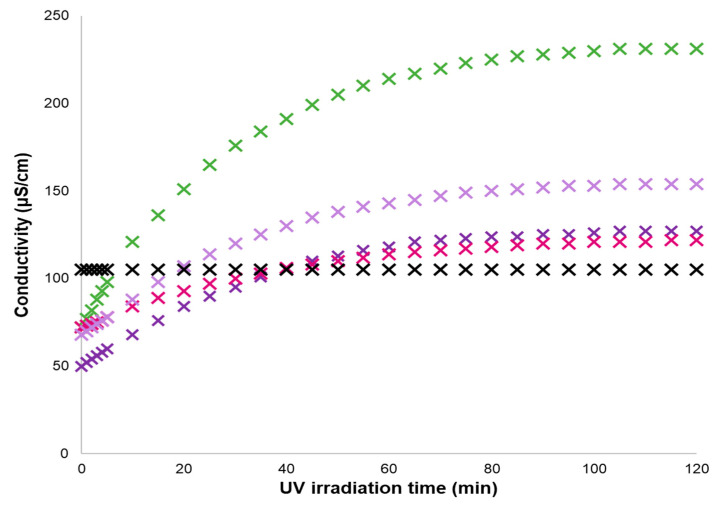
Conductivity data of 6.73 × 10^−4^ mol% IMC1, CH, ET3N, PY and of 1.35 × 10^−3^ mol% KCl aqueous solutions upon UV irradiation time at 25 °C (**X** black cross—KCl, **X** green cross—IMC1, **X** pink cross—CH, **X** lavender cross—ET3N, **X** purple cross—PY).

In Figure 2 and Figure 3 and Table S1 (see Supplementary Information), we present the conductivity data of equimolar (6.73 × 10^−4^ mol%) GDILs aqueous solutions as a function of the irradiation time at 302 nm (formation of the closed form of GDILs, Scheme 4). For comparison purposes, a common salt, KCl, was used. To keep the same chloride anion content, we used a KCl concentration that doubles the one of the GDILS. Unfortunately, we are not able to measure the conductivity contribution of non-ionic photochromic molecules in water solution since these compounds are mainly known for their water insolubility. These studies were used to evaluate the influence of the formation of the closed form GDILs which is controlled by the UV exposure time at 25 °C. We analysed and compared the results using two distinct approaches: one using the same structural cationic moiety, to evaluate the influence of the lateral alkyl chain length of IMCn (Cn = C1, C2, C4, C6, C8 and BENZ) series; another using different cationic motifs, such as IMC1, CH, ET3N and PY.

In the first case (see Figure 2), the initial (open form) conductivity datapoints follow the order: IMC4 ≈ IMC6 ≈ IMC8 > IMC1 ≈ IMC2 ≈ IMBz. The photochromic conversion of GDILs, from the open to the closed form, enhances the solution’s conductivity. The maximum conductivity of GDILs solutions after irradiation follows the order: IMC2 > IMC1 > IMC4 > IMC8 > IMC6 > IMBENZ.

Figure 3 shows that, when distinct cationic moieties are used, the initial (open form) conductivity datapoints follow the order: IMC1 ≈ CH ≈ Et3N > PY. After irradiation (closed form) the maximum conductivity decreases in the order: IMC1 > ET3N > PY > CH.

As expected, the conductivity of a KCl aqueous solution is not affected by UV irradiation and is represented in Figure 2 and Figure 3 as a black cross. Summarizing, the conductivity of a KCl aqueous solution is higher than the aqueous solutions of the corresponding open form of the GDILs. In contrast, photoconversion of GDILs to the closed form strongly increases the conductivity and all GDILs aqueous solutions present values higher than the ones of the KCl solution in water. Even at these extremely low concentrations (6.73 × 10^−4^ mol%), GDILs can increase in almost 240% the solutions’ conductivity, only by UV photoirradiation. These results show that UV photoirradiation can be used to mimic an increase of salt concentration in solution.

This is an interesting result; however, due to the low conductivity of aqueous solutions at these very small salt concentrations, it represents an increase of less than two hundred μScm^−1^. Moreover, in a previous work, we have shown that ionic liquid environments can greatly improve photoconvertability rates of photochromic molecules [38]. With this in mind, we sought to determine the influence of photoisomerization of these gemini photochromic salts in the conductivity of common ionic liquids. The ionic liquids chosen were 1-ethyl-3-methylimidazolium thiocyanate ([C_2_mim][SCN]) and 1-ethyl-3-methylimidazolium bis(trifluoromethanesulfonyl)imide ([C_2_mim][NTf_2_]) which have conductivities on the order of 10–20 mScm^−1^.

We started by determining the maximum solubility of the open form GDILs solubility in this the 1-ethyl-3-methylimidazolium thiocyanate ([C_2_mim][SCN]) (the most hydrophilic ionic liquid). The results are presented in Table 2.

**Scheme 5:**
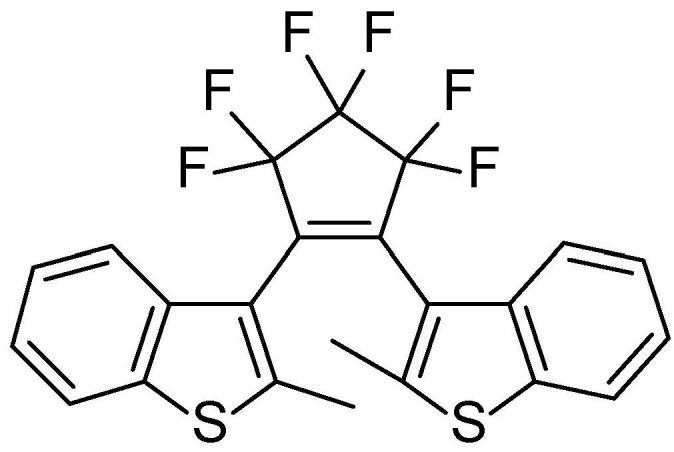
Molecular structure of BTF6.

**Figure 4:**
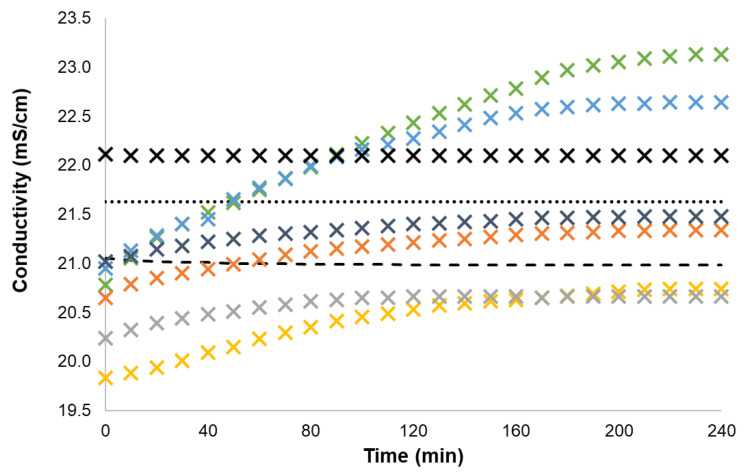
Conductivity data of 0.04 mol% GDIL IMCn series and BTF6, and of 0.08 mol% KCl in [C_2_mim][SCN] solution upon UV irradiation time at 25 °C (round dot—pure [C_2_mim][SCN], dash—BTF6, **X** black cross—KCl, **X** green cross—IMC1, **X** blue cross—IMC2, **X** gold cross—IMC4, **X** gray cross—IMC6, **X** orange cross—IMC8, **X** blue-gray cross—IMBENZ).

GDILs have notoriously higher solubility in [C_2_mim][SCN] than in water. As in aqueous media, the solubility limit of PY is the lowest one at 0.04 mol%. For the photoinduction conductivity studies of GDILs in [C_2_mim][SCN], we used a 0.04 mol% concentration for all GDILs tested. We have also used KCl and BTF6 (Scheme 5) [43] as model compounds for a salt and a photochromic molecular compound, respectively. Again, in the case of KCl, to overcome the effect of the dianionic nature of GDILs (2 anions per each cation), we used a 0.08 mol% concentration of salt. Figure 4 presents the conductivity of imidazolium based GDILs in [C_2_mim][SCN] solution over time of UV irradiation at 25 °C. The experimental data is shown in Supplementary Information (SI)—Section S1, Tables S2–S5.

In Figure 4, for the same cationic moiety (imidazolium) we can observe that all GDILs and BTF6 solutions present lower conductivities than the pure ionic liquid. In contrast, the dissolution of KCl in [C_2_mim][SCN] induces an increase in the solution’s conductivity. In all GDIL cases, there is an increase in conductivity with UV irradiation. This behaviour is similar to the one in aqueous solution. More specifically, IMC1 and IMC2 show the largest increment in conductivity after photoirradiation, surpassing both pure ionic liquid and KCl solution. The conductivity of the closed form GDIL solutions decreases in the order: IMC1 > IMC2 > IMBENZ > IMC8 > IMC4 ≈ IMC6. A very small decrease on conductivity was observed due to the photoirradiation of the BTF6 solution. As expected, the conductivity of the KCl solution did not change upon UV irradiation.

**Figure 5:**
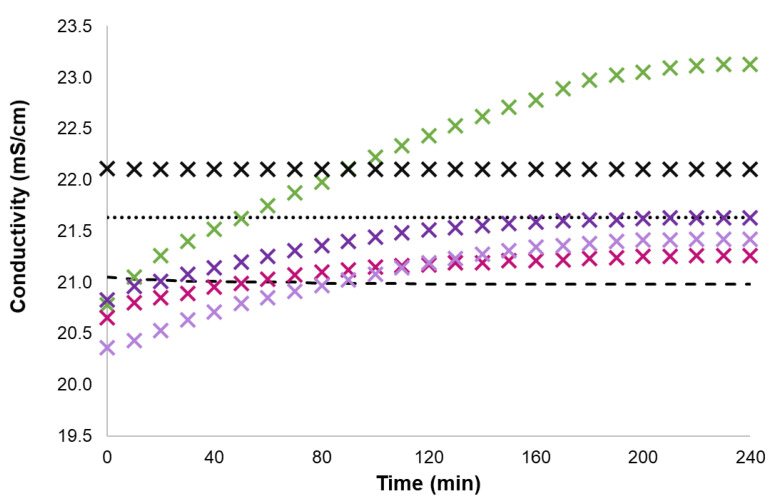
Conductivity data of 0.04 mol% IMC1, CH, ET3N, PY and of 0.08 mol% KCl in [C_2_mim][SCN] solution upon UV irradiation time at 25 °C (round dot—pure [C_2_mim][SCN], dash—BTF6, **X** black cross—KCl, **X** green cross—IMC1, **X** pink cross—CH, **X** lavender cross—ET3N, **X** purple cross—PY).

Figure 5 shows the conductivity data as a function of UV irradiation time for different cationic species, such as IMC1, PY, CH and ET3N. We can observe that before irradiation, all GDILs and BTF6 solutions present lower conductivities than the pure ionic liquid and KCl solution. IMC1 and PY solutions present almost the same initial conductivity value, while for the two non-aromatic ammoniums the value is lower. After irradiation, when the photochromic moiety changes from the open to the closed form, there is always an increase in the solution’s conductivity. In contrast to IMC1, the increment in PY, ET3N and CH is significantly smaller. For different cationic species in [C_2_mim][SCN], the conductivity after irradiation decreases in the order: IMC1 > PY > ET3N > CH.

The main conclusion from the presented data is that, even at very low concentration, GDILs can significantly change the conductivity of ionic liquid solutions. Moreover, the impact on conductivity depends clearly on the cationic moiety connected to the photochromic core centre. The most relevant result was obtained for IMC1 with a clear increase on ionic liquid conductivity of over 2 mScm^−1^ when forcing the closure of the ring of the photochromic centre by UV irradiation. The presence of a hydroxyl functional group in CH compared to ET3N reduces the conductivity contribution of the closed form of the photochromic centre. Interestingly, when using distinct cations, the results in [C_2_mim][SCN] solution follow the trends observed for aqueous solutions.

As the effect observed was similar in water and in a hydrophilic ionic liquid, [C_2_mim][SCN], we have chosen to perform the same experiments in an hydrophobic ionic liquid, 1-ethyl-3-methylimidazolium bis(trifluoromethanesulfonyl)imide ([C_2_mim][NTf_2_]), which is known to be less conductive and more viscous than [C_2_mim][SCN]. To allow the direct comparison of the experimental results, we have used the same GDILs molar concentration as in the experiments with [C_2_mim][SCN]. We have confirmed that all GDILs dissolve completely in [C_2_mim][NTf_2_] at 0.04% molar concentration.

**Figure 6:**
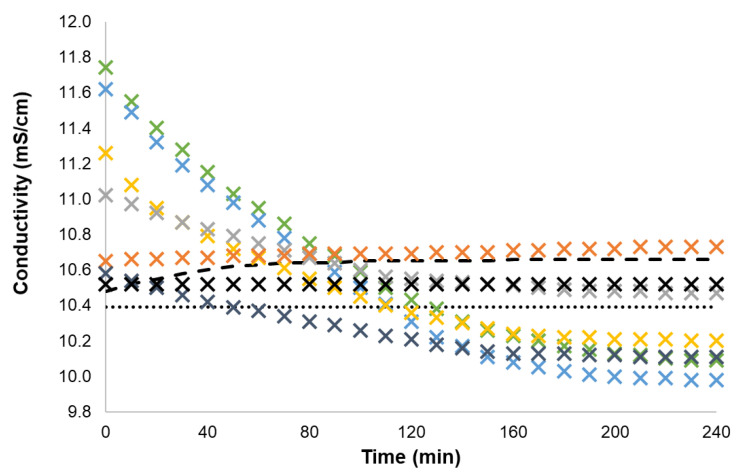
Conductivity data of 0.04 mol% GDIL IMCn series and of 0.08 mol% KCl in [C_2_mim][NTf_2_] solutions upon UV irradiation time at 25 °C (round dot—pure [C_2_mim][NTf_2_], dash—BTF6, **X** black cross—KCl, **X** green cross—IMC1, **X** blue cross—IMC2, **X** gold cross—IMC4, **X** gray cross—IMC6, **X** orange cross—IMC8, **X** blue-gray cross—IMBENZ).

**Figure 7:**
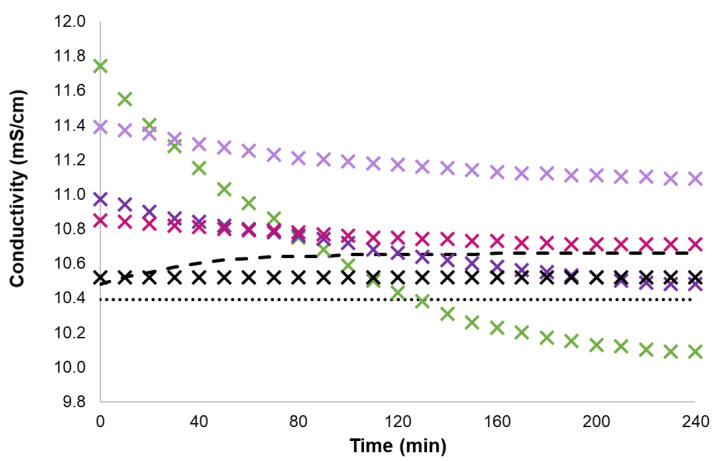
Conductivity data of 0.04 mol% IMC1, CH, ET3N, PY and of 0.08 mol% KCl in [C_2_mim][NTf_2_] solution upon UV irradiation time at 25 °C (round dot—[C2mim][NTf2], dash—BTF6, **X** black cross—KCl, **X** green cross—IMC1, **X** pink cross—CH, **X** lavender cross—ET3N, **X** purple cross—PY).

Figure 6 and Figure 7 present the experimental conductivity data for GDILs solutions in [C_2_mim][NTf_2_] as a function of the UV exposure time at 25 °C.

In Figure 6, the results clearly show that the addition of GDILs in the open form (prior to UV irradiation) to [C_2_mim][NTf_2_] has a positive effect on conductivity, even surpassing the data for the KCl solution. This is precisely the opposite effect observed with GDILs solutions in [C_2_mim][SCN]. Moreover, for the same cationic motifs, IMCn, when we irradiate the solution over time, the conductivity decreases sharply and, in most cases, reaches conductivity values below the one of the pure [C_2_mim][NTf_2_]. The outlier is IMC8, which shows a slight increase in conductivity after irradiation, following the behaviour of the non-ionic photochromic molecule BTF6 (see Supplementary Information, Section S1, Tables S6–S9). For these GDIL systems in [C_2_mim][NTf_2_], the maximum conductivity is obtained for the open form of the photochromic core centre and decreases in order: IMC1 > IMC2 > IMC4 > IMC6 > IMC8 > IMBENZ. Figure 7 shows that the behaviour of GDILs with different cationic species (PY, CH and ET3N) follow the same pattern as the one shown before for IMCn systems. However, the decrease in conductivity is less pronounced and the closed form GDILs solutions conductivity is higher than the one of the pure [C_2_mim][NTf_2_]. The maximum conductivity in [C_2_mim][NTf_2_], is obtained before irradiation and decreases in order: IMC1 > ET3N > PY > CH.

In [C_2_mim][NTf_2_], all the systems show a decrease in conductivity after the photoirradiation process except IMC8 and BTF6. The decrease is smaller with the increase in the alkyl chain length of IMCn. Moreover, the addition of a hydroxyl group to an ammonium moiety (ET3N > CH) reduces the change in the conductivity after irradiation.

These results are striking since they are almost the mirror image of the ones obtained in [C_2_mim][SCN]. Taking into account that the behaviour of GDILs in aqueous solution is similar to the one in [C_2_mim][SCN], we can effectively link the different behaviour of GDILs in solution to the hydrophilic/hydrophobic nature of the media used.

Since conductivity is highly influenced by the properties of the solution, it is relevant to evaluate the viscosity and density changes upon irradiation for all these systems.

### 2.2. Influence of GDILs Photoconversion in the Viscosity of Common Ionic Liquids

Viscosity studies of different GDILs solutions in the two ionic liquids used along this work were performed. In general, conductivity is inversely proportional to viscosity. With these measurements, we want to clarify if the conductivity changes are only related to the viscosity variation in solution or if there are other aspects to consider.

**Figure 8:**
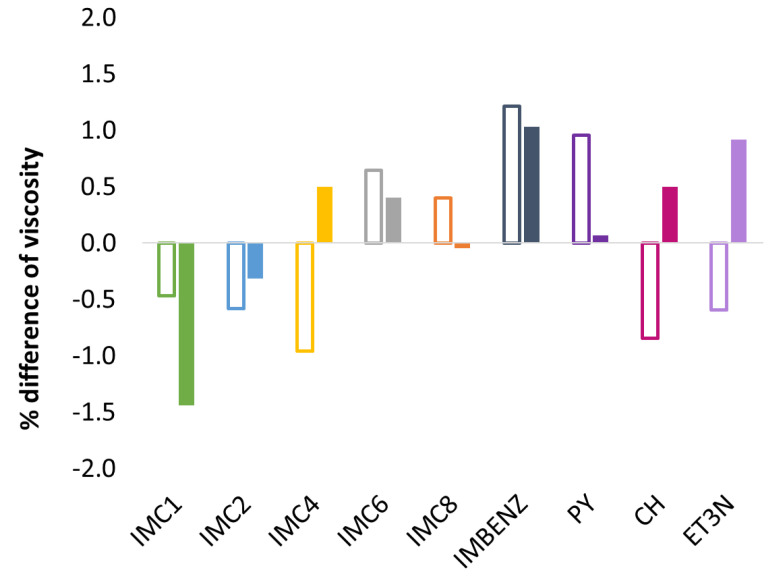
Δ viscosity (in %) of GDILs in [C_2_mim][SCN] solution compared to the pure ionic liquid at 25 °C. Open bars—open form GDIL (no irradiation) and full bars—closed form GDIL (upon UV irradiation, 240 min).

**Figure 9:**
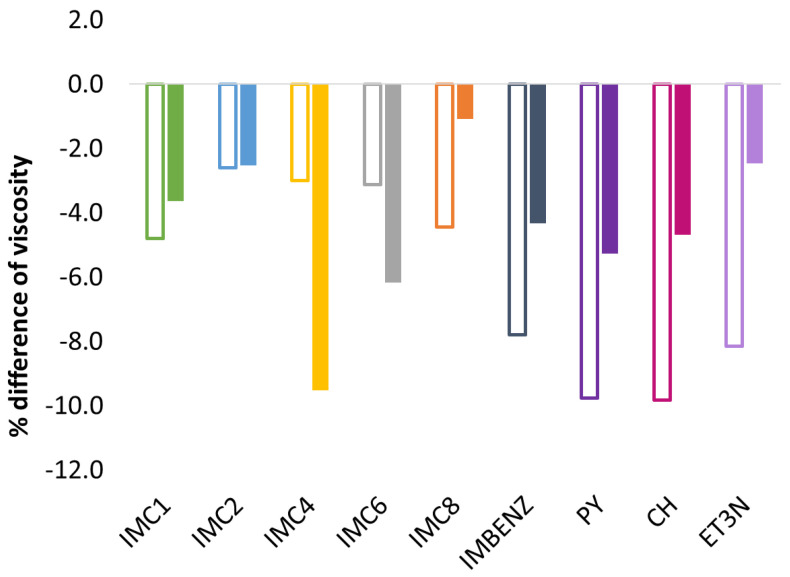
Δ viscosity (in %) of GDILs in [C_2_mim][NTf_2_] solution compared to the pure ionic liquid at 25 °C. Open bars—open form GDIL (no irradiation) and full bars—closed form GDIL (upon UV irradiation, 240 min).

The experimental results are shown in Supplementary Information, Section S2, Tables S10–S13. To facilitate the comprehension of the data, Figure 8 and Figure 9 present the percentual change in viscosity of the GDILs solutions compared with the pure ionic liquid, [C_2_mim][SCN] and [C_2_mim][NTf_2_], respectively. The open bars represent the effect of the dissolution of the open-form GDIL (no irradiation) on the viscosity, while the full bars stand for the closed form GDILs (upon 240 min UV irradiation) effect.

Figure 8 shows no significant changes of viscosity in [C_2_mim][SCN] solutions either by dissolution of the GDIL or its photoirradiation. The differences observed are very close to the expected experimental error (1–2%) of these measurements. Moreover, there is no correlation between the changes in viscosity and conductivity observed.

As shown in Figure 9, the changes obtained for the viscosity of GDILs solutions in [C_2_mim][NTf_2_], are substantially higher than those in [C_2_mim][SCN] solutions. There is a decrease in viscosity by the addition of GDILs (open form) to [C_2_mim][NTf_2_], followed by a slight increase (smaller decrease of the full bar) upon UV irradiation of the GDIL (closed form). The exceptions are IMC4 and IMC6, which present precisely the opposite behaviours. Correlating these results with those of conductivity, the highest effect on conductivity is for IMC1 solution which shows a small variation in viscosity upon irradiation (difference between open and closed bar), while for CH solution, the effect on conductivity is almost null but there is a significant change in the viscosity. One interesting fact that deserves further study in the future is the significant effect of GDILs in the viscosity of [C_2_mim][NTf_2_] solution at the low concentrations used along the work.

### 2.3. Influence of GDILs Photoconversion in the Density of Common Ionic Liquids

Density studies of different GDILs in [C_2_mim][SCN] and [C_2_mim][NTf_2_] solutions were performed. The results obtained show that, at this low concentration, the effect of GDILs addition to ionic liquids is almost negligible. All results are shown in Supplementary Information, Section S3, Tables S14–S17.

## 3. Materials and Methods

### 3.1. Materials

Diethyl ether, petroleum ether 40/60, hexane, cyclohexane, dimethylformamide, dichloromethane, pentane, methanol, toluene, ethanol, ethyl acetate, solution of *n*-butyllithium 2.5 M in hexanes, methyl iodide, 1-butyl chloride, 1-heptyl chloride, 3,4-dihydro-2H-pyran, 1-methylimidazole, diethylethanolamine, triethylamine, phosphorous oxychloride, bromine, Celite^®^ 545, calcium hydride, sodium sulfate anhydrous, sodium chloride, magnesium sulfate anhydrous, potassium hydroxide, and sodium in kerosene were purchased from Sigma-Aldrich with ≥98% purity. Sodium borohydride was obtained from Alfa Aesar with ≥99% purity. Tetrahydrofuran ≥99% purity and silica gel 60, 0.04–0.06 mm for flash chromatography were acquired in Scharlau. Acetic acid (glacial) EMSURE^®^, sulfuric acid 95–97% and TLC silica gel 60 F254 aluminium sheets were purchased in Merck. Pyridine ≥99% purity was obtained from Fluka. Octafluorocyclopentene ≥98% purity was acquired from TCI America. 1-Ethylimidazole, 1-(butyl)imidazole, 1-hexylimidazole, 1-octylimidazole, 1-benzylimidazole were supplied by Iolitec with ≥98% purity. 1,4-dioxane SPECTRANAL ≥99% purity, hydrochloric acid 37% were purchased in Riedel del-Haën. Sodium hydroxide p.a. was acquired from PRONALAB. Diethyl ether, petroleum ether 40/60, hexane, cyclohexane, dimethylformamide, dichloromethane, pentane, methanol, toluene, ethanol, ethyl acetate, 3,4-dihydro-2H-pyran, 1-methylimidazole, 1-ethylimidazole, 1-(butyl)imidazole, 1-hexylimidazole, 1-octylimidazole, 1-benzylimidazole, diethylethanolamine, triethylamine, and pyridine were distilled before use. Tetrahydrofuran, hexane, toluene, methyl iodide, 1-butyl chloride and 1-heptyl chloride were pre-dried in CaH_2_ and refluxed during 72 h with sodium wire under argon atmosphere before use. The 1-ethyl-3-methylimidazolium thiocyanate, [C_2_mim][SCN], and 1-ethyl-3-methylimidazolium bis(trifluoromethanesulfonyl)imide, [C_2_mim][NTf_2_], were purchased from Iolitec with purity > 99 wt%). Potassium chloride (KCl) anhydrous and potassium bis(trifluoromethanesulfonyl)imide (K[NTf_2_]) were acquired from Sigma-Aldrich with a purity of ≥99 wt% and 97 wt%, respectively. Milli Q water was used when necessary to prepare the solutions.

### 3.2. Methods

#### Synthesis and Sample Characterization

##### 2-Methylthiophene (1)

The synthetic procedure of 2-methylthiophene was adapted from the literature [45]. A solution of 2.5 M n-butyllithium (n-BuLi) in hexanes (96 mL; 0.240 mol) was added dropwise, at −78 °C, to 150 mL of a dry tetrahydrofuran (THF) solution of thiophene (19 mL; 0.238 mol) and stirred at that temperature for one hour. A dry THF solution of methyl iodide (14.8 mL; 0.238 mol) was then added dropwise at the previous temperature. After the complete addition, the resultant mixture was slowly warmed to room temperature, and kept stirred overnight. The mixture was then slowly poured into ice cooled water and extracted three times with diethyl ether (Et_2_O). The organic phase was washed several times with water. The organic layer was dried with sodium sulfate (Na_2_SO_4_), filtered, and evaporated. The crude product was purified by column chromatography on silica gel using petroleum ether 40/60 as eluent, to give 1 as a colourless oil (30.7 g; 97% yield). ^1^H-NMR (400 MHz, CDCl_3_): δ = 2.61 (s, 3H, thiophene (2)-CH_3_), 6.87 (d, 1H, J = 3.5 Hz, thiophene (3)-H), 7.01 (d, 1H, J = 3.6 Hz, thiophene (4)-H), 7.18 (d, 1H, J = 3.6 Hz, thiophene (5)-H).

##### 5-Methy-2-formylthiophene (2)

Phosphorous oxychloride (23.7 mL; 0.255 mol) was added dropwise for 30 min to a stirred solution of 1 (10.0 g; 0.102 mol) in 100 mL of dimethylformamide (DMF) at 0 °C [45]. The reaction mixture was warmed to 90 °C for 3 h. The mixture was slowly poured into ice water, neutralized by NaOH, and extracted with dichloromethane. The organic phase was dried over Na_2_SO_4_, filtered and the solvent evaporated. After solvent removal, the product was purified by distillation (30 °C, 0.25 mmHg) giving 2 as a colourless to slightly yellow oil (11.2 g; 87% yield). ^1^H-NMR (400 MHz, CDCl_3_): δ = 2.56 (s, 3H, thiophene (5)-CH_3_), 6.87 (d, 1H, J = 3.52 Hz, thiophene (4)-H), 7.58 (d, 1H, J = 3.72 Hz, thiophene (3)-H), 9.79 (s, 1H, thiophene (2)-CHO).

##### 3-Bromo-2-methyl-5-formylthiophene (3)

A solution of bromine (4.1 mL; 79.3 mmol) was added dropwise over two hours to a stirred solution of 2 (10.0 g; 79.3 mmol) in 50 mL of acetic acid glacial. After full addition, the reaction was stirred for 24 h at 35 °C [45]. The reaction was quenched by the addition of water. The reaction mixture was neutralized with Na_2_CO_3_ and then extracted with dichloromethane. The solution was dried with Na_2_SO_4_, filtered and the solvent evaporated. The crude product was resolubilized in methanol, filtered and the solvent evaporated to obtain the expected product 3. The product 3 was crystallized in hexane to give dark yellow crystals. (14.1 g; 87% yield). ^1^H-NMR (400 MHz, CDCl_3_): δ = 2.46 (s, 3H, thiophene (2)-CH_3_), 7.59 (s, 1H, thiophene (4)-H), 9.77 (s, 1H, thiophene (5)-CHO).

##### 3-Bromo-2-methyl-5-(hydroxymethyl)thiophene (4)

Sodium borohydride (1.91 g; 30.7 mmol) was added to a stirred solution of 3 (6.0 g; 29.3 mmol) in a mixture with ethanol at 0 °C [45]. After the complete addition, the reaction mixture was warmed to 50 °C to increase the solubility, for two hours. The reaction was quenched with water and extracted with ethyl acetate. The organic layers were washed with a saturated solution of sodium chloride (NaCl), separated, dried with Na_2_SO_4_, filtered and the solvent evaporated. The crude product was resolubilized in methanol, filtered and the solvent evaporated to obtain the expected product without further purification. The product 4 was obtained as a colourless to slightly yellow oil (6.0 g; 99% yield). ^1^H-NMR (400 MHz, CDCl_3_): δ = 2.37 (s, 3H, thiophene (2)-CH_3_), 4.71 (s, 2H, -(CH_2_)-thiophene (5)), 6.81 (s, 1H, thiophene (4)-H).

##### 3-Bromo-2-methyl-5-(methoxy(tetrahydro-2H-pyran-2-yl)thiophene (5)

3,4-Dihydro-2H-pyran (13.2 mL; 144.9 mmol) and p-toluenesulfonic acid (0.14 g; 0.5 mole%) were added to a stirred solution of 4 (5.0 g; 24.1 mmol) in 100 mL of dichloromethane [45]. The reaction mixture was refluxed overnight and washed sequentially three times with 3 M NaOH solution and H_2_O, respectively. The organic layer was separated, dried with Na_2_SO_4_, filtered and the solvent evaporated. The crude product was purified by column chromatography on silica gel using hexane/ethyl acetate (4:1), to obtain 5, a slightly yellow oil (6.9 g; 98% yield). ^1^H-NMR (400 MHz, CDCl_3_): δ = 1.48–1.64 (m, 4H, -(CH_2_)_2_-), 1.68–1.74 (m, 1H, CH_2_-pyran), 1.81–1.85 (m, 1H, CH_2_-pyran), 2.41 (s, 3H, thiophene (2)-CH_3_), 3.50–3.56 (m, 1H, CH_2_-pyran), 3.84–3.90 (m, 1H, CH_2_-pyran), 4.60–4.79 (m, 3H, CH_2_-thiophene(5) + -CH-pyran), 6.70 (s, 1H, thiophene (4)-H).

##### 1,2-Bis{2-methyl-5-[2-methoxy(tetrahydro-2H-pyran]-3-thienyl}perfluorocyclopentene (6)

n-Butyllithium 2.5 M in hexane (2.88 mL, 7.21 mmol) was added dropwise to a solution of 5 (2.0 g, 6.87 mmol) in 50 mL of dry tetrahydrofuran at −78 °C, under argon inert atmosphere. The mixture was kept 30 min at −78 °C, and then a solution of octafluorocyclopentene (0.78 g, 3.43 mmmol) in 10 mL of dry THF was slowly added. After stirring for 60 min at −78 °C, the mixture was allowed to slowly warm to room temperature. The reaction was quenched with H_2_O. The organic layer was extracted with diethyl ether (Et_2_O), dried (Na_2_SO_4_), filtered and the solvent removed by evaporation. The crude product was purified by column chromatography on silica gel using pentane. The isomeric mixture is given as a red oil. To isolate the photochromic active isomer, a second purification is needed. The previous mixture was redissolved in 5 mL of hexane and the solution was photoirradiated with a 302 nm UV light for 20 min. The solution changed colour from a clear to a dark red solution. The photoirradiated solution was then purified by column chromatography on silica gel using pentane, only collecting the coloured fraction of the column elution. The photochromic active isomer 6 was isolated as a viscous oil (1.60 g, 39% yield). ^1^H-NMR (400 MHz, CDCl_3_): δ = 1.38–1.54 (m, 4H, -(CH_2_)_2_-), 1.60–1.72 (m, 1H, CH_2_-pyran), 1.79–1.84 (m, 1H, CH_2_-pyran), 2.16 (s, 3H, thiophene (2)-CH_3_), 3.50–3.58 (m, 1H, CH_2_-pyran), 3.80–3.88 (m, 1H, CH_2_-pyran), 4.60–4.78 (m, 3H, CH_2_-thiophene(5) + -CH-pyran), 6.80 (s, 1H, thiophene (4)-H); ^19^F-NMR (376.3 MHz, D_2_O): −109, −133.

##### 1,2-Bis{2-methyl-5-[2-hydroxymethyl]-3-thienyl}perfluorocyclopentene (7)

Phosphorus oxychloride (3.92 mL, 41.9 mmol) was added dropwise to a stirred solution of 6 (5.0 g, 8.38 mmol) in chloroform. The reaction mixture was stirred for 24 h at room temperature. The reaction mixture was neutralized by Na_2_CO_3_, extracted with dichloromethane, dried with Na_2_SO_4_, filtered and the solvent evaporated. The crude product was purified by column chromatography on silica gel using hexane. The product 7 was isolated as a slightly yellow solid (3.4 g, 96% yield). ^1^H-NMR (400 MHz, CDCl3): 1.88 (s, 3H, thiophene (2)-CH_3_), 4.72 (s, 2H, CH_2_-thiophene(5)), 6.98 (s, 1H, thiophene (4)-H); ^13^C-NMR (100 MHz, CDCl_3_): 144.2, 137.9, 126.8, 123.9, 54.7, 14.3; ^19^F-NMR (376.3 MHz, D_2_O): −109, −133.

##### 1,2-Bis{2-methyl-5-[2-chloromethyl]-3-thienyl}perfluorocyclopentene (8)

Sodium chloride was added (4.1 g, 70.0 mmol) to a stirred solution of 7 (3.0 g, 7.0 mmol) in 50 mL of dichloromethane. Then, sulfuric acid (1.87 mL, 35.0 mmol) was added dropwise and stirred overnight at room temperature. The reaction was quenched by the addition of water. The organic layer was separated and washed several times with water, dried with Na_2_SO_4_, filtered and the solvent evaporated. The crude product was purified by column chromatography on silica gel using hexane. The product 8 was isolated as a slightly yellow solid (2.8 g, 87% yield). ^1^H-NMR (400 MHz, CDCl_3_): 1.91 (s, 3H, thiophene (2)-CH_3_), 4.73 (s, 2H, CH_2_-thiophene(5)), 7.05 (s, 1H, thiophene (4)-H); ^13^C-NMR (100 MHz, CDCl_3_): 143.5, 138.6, 127.1, 124.4, 40.0, 14.5; ^19^F-NMR (376.3 MHz, D_2_O): −109, −133.

##### 1,2-Bis{2-methyl-5-[2-(3-N-methylimidazolium)methyl]-3-thienyl}-perfluorocyclopentene dichloride (IMC1)

The 1-Methylimidazole (0.37 g, 4.51 mmol) was added to a stirred solution of 8 (1.00 g, 2.15 mmol) in 25 mL of acetone, in a pressure tube at room temperature. The reaction mixture was then warmed-up to reflux for 3 days. During this process, the formation of precipitate was observed. The solid was separated and washed with cold acetone, to give the product **IMC1** without further purification as a yellow powder (1.30 g, 96% yield). ^1^H-NMR (400 MHz, D_2_O): 1.90 (s, 3H, thiophene (2)-CH_3_), 3.91 (s, 3H, imidazolium(3)-methyl), 5.55 (s, 2H, CH_2_-thiophene(5)), 7.38 (s, 1H, thiophene (4)-H), 7.48 (d, 2H, J = 4.4 Hz, imidazolium(4) + imidazolium(5), 8.80 (s, 1H, (imidazolium(2)); ^13^C-NMR (100 MHz, D_2_O): 136.1, 134.8, 134.3, 133.2, 131.2, 129.1, 125.4, 107.4, 62.4, 40.2, 15.2; ^19^F-NMR (376.3 MHz, D_2_O): −109, −133.

##### 1,2-Bis{2-methyl-5-[2-(3-N-ethylimidazolium)methyl]-3-thienyl}-perfluorocyclopentene dichloride (IMC2)

The 1-Ethylimidazole (0.43 g, 4.51 mmol) was added to a stirred solution of **8** (1.00 g, 2.15 mmol) in 25 mL of acetone, in a pressure tube at room temperature. The reaction mixture was then warmed-up to reflux for 3 days. During this process, the formation of precipitate was observed. The solid was separated and washed with ethyl acetate, to give the product **IMC2** without further purification as a slightly yellow powder (1.31 g, 93% yield). ^1^H-NMR (400 MHz, D_2_O): 1.51 (t, 3H, J = 7.4 Hz, imidazolium (3)-ethyl), 1.89 (s, 3H, thiophene(2)-CH_3_), 4.25 (q, 2H, J = 7.4 Hz, imidazolium(3)-ethyl), 5.56 (s, 2H, CH_2_-thiophene(5)), 7.38 (s, 1H, thiophene (4)-H), 7.50 (d, 1H, J = 1.7 Hz, imidazolium(4)), 7.55 (d, 1H, J = 1.7 Hz, imidazolium(5)), 8.87 (s, 1H, (imidazolium(2)); ^13^C-NMR (100 MHz, D_2_O): 136.1, 134.6, 134.2, 133.1, 131.4, 129.3, 125.5, 107.5, 62.1, 41.3, 18.4, 15.1; ^19^F-NMR (376.3 MHz, D_2_O): −109, −133.

##### 1,2-Bis{2-methyl-5-[2-(3-N-butylimidazolium)methyl]-3-thienyl}-perfluorocyclopentene dichloride (IMC4)

The 1-Butylimidazole (0.56 g, 4.51 mmol) was added to a stirred solution of **8** (1.00 g, 2.15 mmol) in 25 mL of ethyl acetate, in a pressure tube at room temperature. The reaction mixture was then warmed-up to reflux for 3 days. During this process, the formation of precipitate was observed. The solid was separated and washed with ethyl acetate, to give the product **IMC4** without further purification as a dark yellow powder (1.40 g, 91% yield). ^1^H-NMR (400 MHz, D_2_O): 0.91 (t, 3H, J = 7.2 Hz, imidazolium(3)-butyl), 1.27 (sxt, 2H, J = 7.6 Hz, imidazolium(3)-butyl), 1.86 (s, 3H, thiophene(2)-CH_3_), 4.23 (qt, 2H, J = 7.0 Hz, imidazolium(3)-butyl), 4.82–4.86 (m, 2H, imidazolium(3)-butyl), 5.56 (s, 2H, CH_2_-thiophene(5)), 7.31 (s, 1H, thiophene (4)-H), 7.53 (s, 1H, J = 7.6 Hz, imidazolium(4)), 7.55 (s, 1H, imidazolium(5)), 8.90 (s, 1H, (imidazolium(2)); ^13^C-NMR (100 MHz, D_2_O): 136.1, 134.6, 133.2, 131.1, 129.0, 124.9, 107.8, 62.3, 43.4, 29.1, 18.9, 15.0, 12.4; ^19^F-NMR (376.3 MHz, D_2_O): −109, −133.

##### 1,2-Bis{2-methyl-5-[2-(3-N-hexylimidazolium)methyl]-3-thienyl}-perfluorocyclopentene dichloride (IMC6)

The 1-Hexylimidazole (0.69 g, 4.51 mmol) was added to a stirred solution of **8** (1.00 g, 2.15 mmol) in 25 mL of ethyl acetate, in a pressure tube at room temperature. The reaction mixture was then warmed-up to reflux for 3 days. During this process, the formation of precipitate was observed. The solid was separated and washed with diethyl ether, to give the product **IMC6** without further purification as a slightly brown powder (1.44 g, 87% yield). ^1^H-NMR (400 MHz, D_2_O): 0.76 (t, 3H, J = 6.9 Hz, imidazolium(3)-hexyl), 1.12–1.36 (m, 6H, imidazolium(3)-hexyl), 1.80 (s, 3H, thiophene(2)-CH_3_), 4.24 (t, 2H, J = 7.0 Hz, imidazolium(3)-hexyl), 4.82–4.86 (m, 2H, imidazolium(3)-hexyl), 5.58 (s, 2H, CH_2_-thiophene(5)), 7.31 (s,1H, thiophene (4)-H), 7.46 (d, 2H, J = 5.3 Hz, imidazolium (4) + imidazolium(5)), 8.94 (s, 1H, (imidazolium(2)); ^13^C-NMR (100 MHz, D_2_O): 135.8, 134.1, 132.4, 131.2, 129.2, 125.2, 107.2, 62.3, 43.9, 29.8, 28.6, 23.4, 19.8, 15.2, 12.8; ^19^F-NMR (376.3 MHz, D_2_O): −110, −134.

##### 1,2-Bis{2-methyl-5-[2-(3-N-octylimidazolium)methyl]-3-thienyl}-perfluorocyclopentene dichloride (IMC8)

The 1-Octylimidazole (0.81 g, 4.51 mmol) was added to a stirred solution of **8** (1.00 g, 2.15 mmol) in 25 mL of ethyl acetate, in a pressure tube at room temperature. The reaction mixture was then warmed-up to reflux for 3 days. During this process, the formation of precipitate was observed. The solid was separated and washed with diethyl ether and hot hexane to give the product **IMC8** without further purification as a brown powder (1.53 g, 86% yield). ^1^H-NMR (400 MHz, D_2_O): 0.80 (t, 3H, J = 7.3 Hz, imidazolium(3)-octyl), 1.06–1.28 (m, 8H, imidazolium(3)-octyl), 1.78 (t, 3H, thiophene(2)-CH_3_), 1.82–1.88 (m, 2H, imidazolium(3)-octyl), 4.25 (t, 2H, J = 7.0 Hz, imidazolium(3)-octyl), 4.86–4.92 (m, 2H, imidazolium(3)-octyl), 5.58 (s, 2H, CH_2_-thiophene(5)), 7.48 (s,1H, thiophene (4)-H), 7.51 (s, 1H, J = 5.3 Hz, imidazolium(4)), 7.61 (s, 1H, imidazolium(5)), 8.95 (s, 1H, (imidazolium(2)); ^13^C-NMR (100 MHz, D_2_O): 135.8, 134.1, 132.4, 131.3, 129.2, 125.1, 107.0, 62.2, 43.8, 30.1, 28.8, 26.2, 24.5, 20.1, 15.2, 12.7; ^19^F-NMR (376.3 MHz, D_2_O): −110, −134.

##### 1,2-Bis{2-methyl-5-[2-(3-N-benzylimidazolium)methyl]-3-thienyl}-perfluorocyclopentene dichloride (IMBENZ)

The 1-Benzylimidazole (0.71 g, 4.51 mmol) was added to a stirred solution of **8** (1.00 g, 2.15 mmol) in 25 mL of ethyl acetate, in a pressure tube at room temperature. The reaction mixture was then warmed-up to reflux for 3 days. The solvent was removed. The crude product was washed several times with hot hexane, and diethyl ether to give the product **IMBENZ** without further purification as a dark yellow powder (1.51 g, 90% yield). ^1^H-NMR (400 MHz, D_2_O): 1.70 (s, 3H, thiophene(2)-CH_3_), 5.41 (s, 2H, imidazolium (3)-benzyl), 5.53 (s, 2H, CH_2_-thiophene(5)), 7.28 (s, 1H, thiophene (4)-H), 7.35–7.55 (m, 7H, imidazolium(3)-benzyl + imidazolium(4) + imidazolium(5)), 8.87 s, 1H, imidazolium(2)); ^13^C-NMR (100 MHz, D_2_O): 138.8, 135.8, 134.8, 132.8, 131.2, 130.1, 129.1, 127.4, 125.1, 107.2, 62.2, 56.3, 15.2; ^19^F-NMR (376.3 MHz, D_2_O): −110, −134.

##### 1,2-Bis{2-methyl-5-[2-(pyridinium)methyl]-3-thienyl}perfluorocyclo-pentene dichloride (PY)

Pyridine (0.36 g, 4.51 mmol) was added to a stirred solution of **8** (1.00 g, 2.15 mmol) in 25 mL of ethyl acetate, in a pressure tube at room temperature. The reaction mixture was then warmed-up to reflux for 3 days. During this process, the formation of precipitate was observed. The solid was separated and washed with ethyl acetate to give the product **PY** without further purification as a slightly yellow powder (1.16 g, 87% yield). ^1^H-NMR (400 MHz, D_2_O): 1.87 (s, 3H, thiophene(2)-CH_3_), 5.96 (s, 2H, CH_2_-thiophene(5)), 7.48 (s, 1H, thiophene (4)-H), 8.08 (t, 2H, J = 7.1 Hz, pyridinium), 8.59 (t, 1H, J = 7.6 Hz, pyridinium), 8.91 (d, 2H, J = 5.9 Hz, pyridinium); ^13^C-NMR (100 MHz, D_2_O): 148.3, 144.6, 136.1, 134.8, 134.0, 131.2, 129.1, 127.4, 126.9, 107.2, 62.2, 69.1, 15.2; ^19^F-NMR (376.3 MHz, D_2_O): −109, −133.

##### 1,2-Bis{2-methyl-5-[2-(2-hydroxy ethyl)dimethylammonium)methyl]-3-thienyl}perfluorocyclopentene dichloride (CH)

The 2-(Dimethylamino)ethanol (0.40 g, 4.51 mmol) was added to a stirred solution of **8** (1.00 g, 2.15 mmol) in 25 mL of ethyl acetate, in a pressure tube at room temperature. The reaction mixture was then warmed-up to reflux for 3 days. During this process, the formation of precipitate was observed. The solid was separated and washed with ethyl acetate to give the product **CH** without further purification as a clear powder (1.34 g, 97% yield). ^1^H-NMR (400 MHz, D_2_O): 2.01 (s, 3H, thiophene(2)-CH_3_), 3.17 (s, 6H, cholinium), 3.51–3.56 (m, 2H, cholinium), 4.11–4.16 (m, 2H, cholinium), 4.67 (s, 2H, CH2-thiophene(5)), 7.51 (s, 1H, thiophene (4)-H); ^13^C-NMR (100 MHz, D_2_O): 136.1, 134.8, 134.0, 131.2, 129.1, 107.2, 76.1, 74.8, 73.8, 72.1, 61.3, 15.2; ^19^F-NMR (376.3 MHz, D_2_O): −109, −133.

##### 1,2-Bis{2-methyl-5-[2-(triethylammonium)methyl]-3-thienyl}perfluoro-cyclopentene dichloride (ET3N)

Triethylamine (0.46 g, 4.51 mmol) was added to a stirred solution of **8** (1.00 g, 2.15 mmol) in 25 mL of ethyl acetate, in a pressure tube at room temperature. The reaction mixture was then warmed-up to reflux for 3 days. During this process, the formation of precipitate was observed. The solid was separated and washed with ethyl acetate to give the product **ET3N** without further purification as a slightly yellow powder (1.38 g, 96% yield). ^1^H-NMR (400 MHz, D_2_O): 1.39 (t, 9H, J = 7.1 Hz, triethylammonium), 2.03 (s, 3H, thiophene(2)-CH_3_), 3.29 (q, 6H, triethylammonium), 3.51–3.56 (m, 2H, cholinium(3)), 4.61 (s, 2H, CH_2_-thiophene(5)), 7.43 (s,1H, thiophene (4)-H); ^13^C-NMR (100 MHz, D_2_O): 136.1, 134.8, 134.0, 131.2, 129.1, 107.2, 70.8, 59.4, 15.2, 13.4; ^19^F-NMR (376.3 MHz, D_2_O): −109, −133.

##### ^1^H-,^13^C- and ^19^F-NMR Spectra Were Obtained on an Ultrashield Bruker Avance II 400 Spectrometer: Chemical Shifts Are Reported in Parts per Million

A UVP benchtop UV transilluminator LMS-26, Cambridge, UK, with triple wavelength (254/302/365 nm), single intensity using an 8W output was used in the photoirradiation process of all the samples.

The GDILs solubility in water or ILs was performed at 20 ± 0.5 °C. A stepwise solvent addition technique was used. More specifically, a small volume of solvent (water or ionic liquid) was added to a predefined quantity of GDIL (roughly 0.01 g), in a 10 mL glass fixed sealed tube with a magnetic stirrer, loaded up with argon. After every solvent addition, the blend was mixed and outwardly checked for any undissolved GDIL. Preliminary tests identified the solubility range that would be tested in more rigorous measurements. Each rigorous solubility test was performed in triplicate.

Ionic conductivities were measured using a Mettler Toledo Five Easy Plus (FP30) benchtop meter, Schwerzenbach, Switzerland, with an attached Mettler Toledo InLab^®^ 751-4 mm sensor with an internal temperature control (±0.1 K). The measurements were made within the temperature range of 293.15 to 323.15 K, with a temperature step of 5 K. For high-precision measurements, it was necessary to determine the cell constant by performing calibration measurements on the temperature range 293.15 to 323.15 K, using three calibrating standards, KCl 0.01 D, KCl 0.1 D and KCl 1.0 D solutions. Measurements were performed in a 5 mL glass cell containing a magnetic stirrer and temperature regulated by a water jacket connected to a bath controlled with an uncertainty of 0.01 K. The temperature of the cell was measured using high-accuracy mercury thermometers of different temperature range (0.01 K) [46]. All GDILs were pre-dried under vacuum for 2 weeks, at 60 °C. All GDIL ionic liquids solutions were prepared under argon atmosphere. GDILs were dissolved in water and in different ionic liquids at 3.74 × 10^−4^ mol% and 0.04 mol% concentration, respectively (5 mL solutions). All GDILs ionic liquid solutions were pre-dried under vacuum at 50 °C, for at least one week. Before any conductivity measurements, all solutions were filled with argon. All conductivity measurements were made under argon atmosphere. GDILs solutions conductivities were compared with measured conductivities of the pure solvents. Conductivity measurements were made in triplicates for GDIL + water solutions (before and after photoirradiation at λ = 302 nm up to 2 h) and pure water. Conductivity measurements were made in triplicates for GDIL + ionic liquid solutions (before and after photoirradiation at λ = 302 nm up to 4 h) and pure ionic liquids. The obtained conductivity data, for each sample, in different media, is the average of triplicate measurements.

Measurements of viscosity were performed at atmospheric pressure, in the temperature range of 293.15 K to 373.15 K, with a temperature step of 5 K using an automated SVM 3000 Anton Paar rotational Stabinger viscometer, Graz, Austria. The viscometer was calibrated using specific oils from the supplier and following the procedure described on the equipment manual. The temperature uncertainty is ±0.02 K from 293.15 to 373.15 K. The precision of the dynamic viscosity measurements is ±0.5%. The overall uncertainty of the measurements is estimated to be 2% [47,48,49]. GDILs were dissolved in different ionic liquids at 0.04 mol% concentration (5 mL solutions). All samples were pre-dried under vacuum at 50 °C, for at least 5 days. GDILs solutions viscosities were compared with measured viscosities of the pure ionic liquids. Viscosity measurements were made in triplicate for GDILs + ionic liquids solutions (before and after photoirradiation at λ = 302 nm up to 4 h) and pure ionic liquids. The obtained viscosity data, for each sample, is the average of triplicate measurements.

Density was measured with an Anton Paar vibrating tube densimeter, model DMA 5000, Graz, Austria, operating at atmospheric pressure and in the temperature range 293.15 K to 323.15 K, with a temperature step of 5 K. The densimeter was calibrated by measuring the densities of atmospheric air and Milli Q water, according to the manufacturer’s recommendations. All reported density data were corrected for the effect of viscosity using the internal calibration of the densimeter. Under these operating conditions, we found that the repeatability of the density measurements was better than 0.04 kg·m^−3^ and the expanded uncertainty was estimated to be ±0.3 kg·m^−3^ for the ionic liquids used in this work [50]. For the density measurements, the same sample preparation used in the viscosity measurements was followed.

## 4. Conclusions

The GDILs produced are innovative salts that present photoinduced properties that can alter solution properties such as conductivity and viscosity, by photoirradiation, even at very low concentrations of GDIL in solution. They show a tremendous enhancement on the solubility in water compared to traditional diarylethene-based photochromic compounds. In this work, we have shown that they are also soluble in distinct ionic liquids. The effect in the measured solution properties, either with ionic liquids or water as the solvent, was evaluated in two distinct ways: (i) effect of addition of the GDIL (open form) to the solution and (ii) effect of photoirradiation (closed form). GDILs with imidazolium cation motifs are more susceptible to induce higher changes in solution, followed by non-aromatic ammonium cations.

It is important to stress the opposite behaviour of GDILs in solution in the two distinct ionic liquids tested, [C_2_mim][SCN] and [C_2_mim][NTf_2_]. The addition of GDIL to the [C_2_mim][SCN] decreases the conductivity of the IL and the photoirradiation of the solution increases it. This contrasts with the increase on the conductivity of [C_2_mim][NTf_2_] upon GDIL addition and the concomitant decrease after photoirradiation. From our perspective, this is related to the more hydrophilic/hydrophobic properties of the solvent. IMC1 and IMC2 are the most prone to induce higher changes in the solution’s conductivity by photoirradiation.

The experimental results show that the changes in conductivity are not related to the viscosity or density changes in the solution and, so, further studies must be carried out to explain the observed behaviour.

The possibility of controlling the conductivity of the solutions by UV photoirradiation, can be applied in systems susceptible to these changes, including stereochemistry, [51,52,53] polymorphism controllability, [54,55,56,57] and several other uses in material science, for new innovative materials with specific and/or enhanced properties [58,59,60].

The applications of these gemini diarylethenes-based ionic liquids can be further expanded, since these new salts have higher water solubilities than commonly used photochromic molecules. They also show high photoinduced capabilities to improve and/or change physical and chemical properties in non-ionic and ionic solutions, especially their conductivities and viscosities.

## Figures and Tables

**Table 1:** Maximum solubility, in mass percentage (mass%), mol percentage (mol%) and molar concentration (M), of GDILs in H_2_O.

Acronym	mass%	mol%	[C]/M
IMC1	5.91	1.69 × 10^−1^	9.39 × 10^−2^
IMC2	4.62	1.26 × 10^−1^	7.03 × 10^−2^
IMC4	2.82	7.10 × 10^−2^	3.95 × 10^−2^
IMC6	1.76	4.12 × 10^−2^	2.29 × 10^−2^
IMC8	0.91	1.99 × 10^−2^	1.10 × 10^−2^
IMBENZ	2.82	6.50 × 10^−2^	3.61 × 10^−2^
PY	0.02	6.73 × 10^−4^	3.74 × 10^−4^
CH	31.72	8.87 × 10^−1^	4.93 × 10^−1^
ET3N	13.58	3.66 × 10^−1^	2.03 × 10^−1^

**Table 2:** Maximum solubility, in mass percentage (mass%), mol percentage (mol%) and molar concentration (M), of GDILs in [C_2_mim][SCN].

Acronym	mass%	mol%	[C]/M
IMC1	6.00	1.61	1.06 × 10^−1^
IMC2	4.57	1.18	7.76 × 10^−2^
IMC4	4.26	1.01	6.67 × 10^−2^
IMC6	2.35	0.52	3.40 × 10^−2^
IMC8	2.07	0.42	2.79 × 10^−2^
IMBENZ	2.63	0.57	3.76 × 10^−2^
PY	0.23	0.04	4.11 × 10^−3^
CH	7.00	1.84	1.21 × 10^−1^
ET3N	4.26	1.08	7.31 × 10^−2^

## Data Availability

Not applicable.

## Supplementary Materials

The following supporting information can be downloaded at: https://www.mdpi.com/article/10.3390/ijms24043533/s1.
