# Supplementary material for: Diarylethene-Based Ionic Liquids: Synthesis and Photo-Driven Solution Properties
Source: Int J Mol Sci. 2023 Feb 9;24(4):3533. doi: 10.3390/ijms24043533 (PMC9960670; doi:10.3390/ijms24043533)
Supplement: Supplementary file 1 [file ijms-24-03533-s001.zip › ijms-2158120-supplementary.pdf]

# Diarylethene based Ionic Liquids: Synthesis and Photodriven Solution Properties

Mário R. C. Soromenho[1], Carlos A. M. Afonso[2], José M. S. S. Esperança[1,*]

[1]LAQV-REQUIMTE, Departamento de Química, Faculdade de Ciências e Tecnologia, Universidade NOVA de Lisboa, 2829-516, Caparica, Portugal;

[2]Research Institute for Medicine (iMed.ULisboa); Faculty of Pharmacy, Universidade de Lisboa, Lisboa 1649-003, Portugal.

*Correspondence: jmesp@fct.unl.pt (J.M.S.S.E.)

## SUPPLEMENTARY INFORMATION

## Section 1 – Conductivity studies

**Table S1-1 -** Conductivity evolution of GDILs aqueous solutions over UV irradiation time, at 25ºC.

| | Conductivity ($\mu S.cm^{-1}$) | | | | | | | | | |
|---|---|---|---|---|---|---|---|---|---|---|
| **t** $_{Uv\ irradiation}$ **(min)** / **Solution** | **KCl** | **IMC1** | **IMC2** | **IMC4** | **IMC6** | **IMC8** | **IMBENZ** | **PY** | **CH** | **ET3N** |
| **0** | 105 | 72 | 70 | 93 | 86 | 90 | 66 | 50 | 72 | 68 |
| **1** | 105 | 77 | 74 | 96 | 89 | 93 | 68 | 52 | 73 | 70 |
| **2** | 105 | 82 | 78 | 99 | 92 | 96 | 69 | 54 | 74 | 72 |
| **3** | 105 | 88 | 82 | 102 | 94 | 99 | 72 | 56 | 75 | 74 |
| **4** | 105 | 93 | 89 | 105 | 96 | 102 | 77 | 58 | 76 | 76 |
| **5** | 105 | 98 | 95 | 109 | 99 | 104 | 81 | 60 | 78 | 78 |
| **10** | 105 | 121 | 119 | 123 | 109 | 114 | 91 | 68 | 84 | 88 |
| **15** | 105 | 136 | 135 | 134 | 118 | 124 | 100 | 76 | 89 | 98 |
| **20** | 105 | 151 | 153 | 148 | 127 | 132 | 108 | 84 | 93 | 107 |
| **25** | 105 | 165 | 168 | 158 | 133 | 142 | 116 | 90 | 97 | 114 |
| **30** | 105 | 176 | 180 | 168 | 139 | 150 | 122 | 95 | 100 | 120 |
| **40** | 105 | 184 | 191 | 178 | 145 | 158 | 128 | 101 | 103 | 125 |
| **45** | 105 | 191 | 199 | 186 | 150 | 167 | 134 | 106 | 106 | 130 |
| **50** | 105 | 199 | 208 | 193 | 153 | 174 | 138 | 110 | 108 | 135 |
| **55** | 105 | 205 | 215 | 199 | 156 | 180 | 142 | 113 | 110 | 138 |
| **60** | 105 | 210 | 220 | 204 | 160 | 185 | 146 | 116 | 112 | 141 |
| **65** | 105 | 214 | 224 | 209 | 163 | 189 | 148 | 118 | 114 | 143 |
| **70** | 105 | 217 | 227 | 212 | 165 | 192 | 151 | 121 | 115 | 145 |
| **75** | 105 | 220 | 230 | 214 | 168 | 194 | 154 | 122 | 116 | 147 |
| **80** | 105 | 223 | 232 | 217 | 170 | 196 | 156 | 123 | 117 | 149 |
| **85** | 105 | 225 | 233 | 219 | 171 | 197 | 159 | 124 | 118 | 150 |
| **90** | 105 | 227 | 234 | 221 | 172 | 198 | 161 | 124 | 119 | 151 |
| **95** | 105 | 228 | 235 | 223 | 173 | 199 | 163 | 125 | 120 | 152 |
| **100** | 105 | 229 | 235 | 224 | 174 | 199 | 165 | 125 | 120 | 153 |
| **105** | 105 | 230 | 236 | 225 | 175 | 200 | 166 | 126 | 121 | 153 |
| **110** | 105 | 231 | 236 | 226 | 176 | 200 | 167 | 127 | 121 | 154 |
| **115** | 105 | 231 | 236 | 226 | 176 | 201 | 167 | 127 | 121 | 154 |
| **120** | 105 | 231 | 237 | 227 | 177 | 201 | 168 | 127 | 122 | 154 |

**Table S1-2 -** Conductivity of pure [$C_2$mim][SCN] and GDIL/[$C_2$mim][SCN] solutions before UV irradiation, at different temperatures.

| | Conductivity (mS.cm$^{-1}$) | | | | | | | | | |
|---|---|---|---|---|---|---|---|---|---|---|
| **Solution / T (ºC)** | **[$C_2$mim][SCN]** | **IMC1** | **IMC2** | **IMC4** | **IMC6** | **IMC8** | **IMBENZ** | **PY** | **CH** | **ET3N** |
| **20** | 18.50 | 17.61 | 17.82 | 16.75 | 17.15 | 17.59 | 17.92 | 17.7 | 17.55 | 17.15 |
| **25** | 21.60 | 20.78 | 20.95 | 19.83 | 20.24 | 20.65 | 21.02 | 20.83 | 20.65 | 20.36 |
| **30** | 25.09 | 24.24 | 24.42 | 23.29 | 23.69 | 24.06 | 24.45 | 24.29 | 24.24 | 23.80 |
| **35** | 28.67 | 27.82 | 27.94 | 26.90 | 27.31 | 27.73 | 28.06 | 27.87 | 27.83 | 27.39 |
| **40** | 32.39 | 31.54 | 31.68 | 30.72 | 31.00 | 31.32 | 31.77 | 31.59 | 31.55 | 31.15 |
| **45** | 36.32 | 35.47 | 35.59 | 34.55 | 35.02 | 35.25 | 35.69 | 35.52 | 34.48 | 35.06 |
| **50** | 40.52 | 39.67 | 39.82 | 38.87 | 39.15 | 39.54 | 39.91 | 39.72 | 39.68 | 39.26 |

**Table S1-3 -** Conductivity of pure [$C_2$mim][SCN] pure and GDIL/[$C_2$mim][SCN] solutions after UV irradiation, at different temperatures.

| | Conductivity (mS.cm$^{-1}$) | | | | | | | | | |
|---|---|---|---|---|---|---|---|---|---|---|
| **Solution / T (ºC)** | **[$C_2$mim] [SCN]** | **IMC1** | **IMC2** | **IMC4** | **IMC6** | **IMC8** | **IMBENZ** | **PY** | **CH** | **ET3N** |
| **20** | 18.50 | 20.01 | 19.51 | 17.64 | 17.41 | 18.29 | 18.32 | 18.5 | 18.15 | 18.27 |
| **25** | 21.60 | 23.13 | 22.64 | 20.74 | 20.66 | 21.34 | 21.48 | 21.63 | 21.26 | 21.42 |
| **30** | 25.09 | 26.59 | 26.1 | 24.2 | 24.52 | 24.76 | 24.89 | 25.09 | 24.72 | 24.89 |
| **35** | 28.67 | 30.17 | 29.68 | 27.74 | 28.08 | 28.53 | 28.49 | 28.67 | 28.28 | 28.42 |
| **40** | 32.39 | 33.89 | 33.4 | 31.46 | 31.77 | 32.06 | 32.25 | 32.39 | 31.97 | 32.08 |
| **45** | 36.32 | 37.82 | 37.33 | 35.33 | 35.68 | 35.99 | 36.18 | 36.32 | 35.88 | 35.98 |
| **50** | 40.52 | 42.03 | 41.53 | 39.53 | 39.84 | 40.2 | 40.39 | 40.52 | 40.04 | 40.13 |

**Table S1-4 -** Conductivity evolution of pure [$C_2$mim][SCN], KCl and BTF6 solutions in [$C_2$mim][SCN] over UV irradiation time, at 25ºC

| | Conductivity ($mS.cm^{-1}$) | | |
|---|---|---|---|
| Solution / $t_{Uv\ irradiation}$ (min) | [$C_2$mim][SCN] | KCl | BTF6 |
| **0** | 21.60 | 22.11 | 21.05 |
| **10** | 21.60 | 22.10 | 21.03 |
| **20** | 21.60 | 22.10 | 21.02 |
| **30** | 21.60 | 22.10 | 21.01 |
| **40** | 21.60 | 22.10 | 21.01 |
| **50** | 21.60 | 22.10 | 21.00 |
| **60** | 21.60 | 22.10 | 21.00 |
| **70** | 21.60 | 22.10 | 21.00 |
| **80** | 21.60 | 22.10 | 20.99 |
| **90** | 21.60 | 22.10 | 20.99 |
| **100** | 21.60 | 22.10 | 20.99 |
| **110** | 21.60 | 22.11 | 20.99 |
| **120** | 21.60 | 22.10 | 20.98 |
| **130** | 21.60 | 22.10 | 20.98 |
| **140** | 21.60 | 22.10 | 20.98 |
| **150** | 21.60 | 22.10 | 20.98 |
| **160** | 21.60 | 22.10 | 20.98 |
| **170** | 21.60 | 22.10 | 20.98 |
| **180** | 21.60 | 22.10 | 20.98 |
| **190** | 21.60 | 22.10 | 20.98 |
| **200** | 21.60 | 22.10 | 20.98 |
| **210** | 21.60 | 22.10 | 20.98 |
| **220** | 21.60 | 22.10 | 20.98 |
| **230** | 21.60 | 22.10 | 20.98 |
| **240** | 21.60 | 22.10 | 20.98 |

**Table S1-5 -** Conductivity evolution of GDIL/[$C_2$mim][SCN] solutions over UV irradiation time, at 25ºC

| | Conductivity ($mS.cm^{-1}$) | | | | | | | | |
|---|---|---|---|---|---|---|---|---|---|
| Solution / $t_{Uv\ irradiation}$ (min) | IMC1 | IMC2 | IMC4 | IMC6 | IMC8 | IMBENZ | PY | CH | ET3N |
| **0** | 20.78 | 20.95 | 19.83 | 20.24 | 20.65 | 21.02 | 20.83 | 20.65 | 20.36 |
| **10** | 21.05 | 21.13 | 19.88 | 20.32 | 20.79 | 21.07 | 20.96 | 20.80 | 20.43 |
| **20** | 21.26 | 21.28 | 19.94 | 20.39 | 20.85 | 21.14 | 21.01 | 20.85 | 20.53 |
| **30** | 21.40 | 21.40 | 20.01 | 20.44 | 20.90 | 21.18 | 21.08 | 20.89 | 20.63 |
| **40** | 21.52 | 21.45 | 20.09 | 20.48 | 20.94 | 21.22 | 21.14 | 20.95 | 20.71 |
| **50** | 21.62 | 21.65 | 20.15 | 20.51 | 20.99 | 21.25 | 21.20 | 20.99 | 20.79 |
| **60** | 21.75 | 21.77 | 20.23 | 20.55 | 21.04 | 21.28 | 21.25 | 21.03 | 20.85 |
| **70** | 21.87 | 21.86 | 20.29 | 20.58 | 21.09 | 21.30 | 21.31 | 21.07 | 20.91 |
| **80** | 21.98 | 21.99 | 20.35 | 20.61 | 21.12 | 21.32 | 21.36 | 21.10 | 20.97 |
| **90** | 22.11 | 22.08 | 20.41 | 20.63 | 21.15 | 21.34 | 21.40 | 21.12 | 21.02 |
| **100** | 22.22 | 22.16 | 20.45 | 20.65 | 21.17 | 21.36 | 21.44 | 21.15 | 21.08 |
| **110** | 22.33 | 22.21 | 20.49 | 20.65 | 21.19 | 21.38 | 21.48 | 21.16 | 21.14 |
| **120** | 22.43 | 22.27 | 20.53 | 20.66 | 21.21 | 21.40 | 21.51 | 21.17 | 21.19 |
| **130** | 22.53 | 22.34 | 20.57 | 20.66 | 21.23 | 21.41 | 21.53 | 21.19 | 21.23 |
| **140** | 22.62 | 22.41 | 20.59 | 20.66 | 21.25 | 21.42 | 21.55 | 21.19 | 21.27 |
| **150** | 22.71 | 22.48 | 20.61 | 20.66 | 21.27 | 21.43 | 21.57 | 21.21 | 21.31 |
| **160** | 22.78 | 22.53 | 20.63 | 20.66 | 21.29 | 21.45 | 21.59 | 21.21 | 21.34 |
| **170** | 22.89 | 22.57 | 20.65 | 20.65 | 21.30 | 21.46 | 21.60 | 21.22 | 21.36 |
| **180** | 22.97 | 22.59 | 20.67 | 20.66 | 21.31 | 21.46 | 21.61 | 21.23 | 21.38 |
| **190** | 23.02 | 22.61 | 20.69 | 20.66 | 21.32 | 21.47 | 21.61 | 21.24 | 21.40 |
| **200** | 23.05 | 22.63 | 20.71 | 20.66 | 21.33 | 21.47 | 21.62 | 21.25 | 21.41 |
| **210** | 23.09 | 22.63 | 20.73 | 20.66 | 21.33 | 21.48 | 21.63 | 21.25 | 21.41 |
| **220** | 23.11 | 22.64 | 20.74 | 20.66 | 21.34 | 21.48 | 21.63 | 21.26 | 21.42 |
| **230** | 23.13 | 22.64 | 20.74 | 20.66 | 21.34 | 21.48 | 21.63 | 21.26 | 21.42 |
| **240** | 23.13 | 22.64 | 20.74 | 20.66 | 21.34 | 21.48 | 21.63 | 21.26 | 21.42 |

**Table S1-6 -** Conductivity of pure [$C_2$mim][$NTf_2$] and GDIL/[$C_2$mim][$NTf_2$] solutions before UV irradiation, at different temperatures.

| | Conductivity (mS.cm$^{-1}$) | | | | | | | | | |
|---|---|---|---|---|---|---|---|---|---|---|
| T (ºC) \ Solution | [$C_2$mim][$NTf_2$] | IMC1 | IMC2 | IMC4 | IMC6 | IMC8 | IMBENZ | PY | CH | ET3N |
| **20** | 8.82 | 10.08 | 9.939 | 9.638 | 9.389 | 9.05 | 8.988 | 9.334 | 9.253 | 9.734 |
| **25** | 10.39 | 11.74 | 11.62 | 11.26 | 11.02 | 10.65 | 10.58 | 10.97 | 10.85 | 11.39 |
| **30** | 12.11 | 13.55 | 13.39 | 13.02 | 12.76 | 12.4 | 12.3 | 12.72 | 12.54 | 13.15 |
| **35** | 14.15 | 15.48 | 15.25 | 14.86 | 14.59 | 14.28 | 14.2 | 14.54 | 14.44 | 14.98 |
| **40** | 16.22 | 17.51 | 17.29 | 16.79 | 16.58 | 16.34 | 16.07 | 16.6 | 16.34 | 16.88 |
| **45** | 18.44 | 19.55 | 19.24 | 18.76 | 18.63 | 18.44 | 18.22 | 18.6 | 18.46 | 18.91 |
| **50** | 20.7 | 21.8 | 21.47 | 20.98 | 20.83 | 20.67 | 20.41 | 20.71 | 20.66 | 21.05 |

**Table S1-7 -** Conductivity of pure [$C_2$mim][$NTf_2$] and GDIL/[$C_2$mim][$NTf_2$] solutions after UV irradiation, at different temperatures.

| | Conductivity (mS.cm$^{-1}$) | | | | | | | | | |
|---|---|---|---|---|---|---|---|---|---|---|
| T (ºC) \ Solution | [$C_2$mim][$NTf_2$] | IMC1 | IMC2 | IMC4 | IMC6 | IMC8 | IMBENZ | PY | CH | ET3N |
| **20** | 8.82 | 8.542 | 8.135 | 8.297 | 8.482 | 8.887 | 8.349 | 8.953 | 9.107 | 9.43 |
| **25** | 10.39 | 10.09 | 9.978 | 10.2 | 10.47 | 10.73 | 10.11 | 10.48 | 10.71 | 11.09 |
| **30** | 12.11 | 11.64 | 11.78 | 12.17 | 12.51 | 12.86 | 11.85 | 12.22 | 12.43 | 12.75 |
| **35** | 14.15 | 13.22 | 13.74 | 14.38 | 14.79 | 15.19 | 13.71 | 14.71 | 14.33 | 14.57 |
| **40** | 16.22 | 14.76 | 15.61 | 16.59 | 17.05 | 17.42 | 15.66 | 16.23 | 15.93 | 16.36 |
| **45** | 18.44 | 16.32 | 17.6 | 18.88 | 19.34 | 19.75 | 16.95 | 18.49 | 18.06 | 18.41 |
| **50** | 20.70 | 17.89 | 19.59 | 21.29 | 21.6 | 22.09 | 19.47 | 20.71 | 20.28 | 20.51 |

**Table S1-8 -** Conductivity evolution of pure $[C_2mim][NTf_2]$, KCl and BTF6 solutions in $[C_2mim][NTf_2]$ over UV irradiation time, at 25ºC

| | **Conductivity (mS.cm$^{-1}$)** | | |
|---|---|---|---|
| **Solution** / **t** $_{Uv\ irradiation}$ **(min)** | **$[C_2mim][NTf_2]$** | **KCl** | **BTF6** |
| **0** | 10.39 | 10.5 | 10.5 |
| **10** | 10.39 | 10.5 | 10.5 |
| **20** | 10.39 | 10.5 | 10.6 |
| **30** | 10.39 | 10.5 | 10.6 |
| **40** | 10.39 | 10.5 | 10.6 |
| **50** | 10.39 | 10.5 | 10.6 |
| **60** | 10.39 | 10.5 | 10.6 |
| **70** | 10.39 | 10.5 | 10.6 |
| **80** | 10.39 | 10.5 | 10.6 |
| **90** | 10.39 | 10.5 | 10.6 |
| **100** | 10.39 | 10.5 | 10.7 |
| **110** | 10.39 | 10.5 | 10.7 |
| **120** | 10.39 | 10.5 | 10.7 |
| **130** | 10.39 | 10.5 | 10.7 |
| **140** | 10.39 | 10.5 | 10.7 |
| **150** | 10.39 | 10.5 | 10.7 |
| **160** | 10.39 | 10.5 | 10.7 |
| **170** | 10.39 | 10.5 | 10.7 |
| **180** | 10.39 | 10.5 | 10.7 |
| **190** | 10.39 | 10.5 | 10.7 |
| **200** | 10.39 | 10.5 | 10.7 |
| **210** | 10.39 | 10.5 | 10.7 |
| **220** | 10.39 | 10.5 | 10.7 |
| **230** | 10.39 | 10.5 | 10.7 |
| **240** | 10.39 | 10.5 | 10.7 |

**Table S1-9 -** Conductivity evolution of GDIL/[$C_2$mim][$NTf_2$] solutions over UV irradiation time, at 25ºC

| | Conductivity (mS.cm$^{-1}$) | | | | | | | | |
|---|---|---|---|---|---|---|---|---|---|
| **Solution / $t_{Uv\ irradiation}$ (min)** | **IMC1** | **IMC2** | **IMC4** | **IMC6** | **IMC8** | **IMBENZ** | **PY** | **CH** | **ET3N** |
| **0** | 11.7 | 11.6 | 11.3 | 11.0 | 10.7 | 10.6 | 11.0 | 10.9 | 11.4 |
| **10** | 11.6 | 11.5 | 11.1 | 11.0 | 10.7 | 10.5 | 10.9 | 10.8 | 11.4 |
| **20** | 11.4 | 11.3 | 11.0 | 10.9 | 10.7 | 10.5 | 10.9 | 10.8 | 11.4 |
| **30** | 11.3 | 11.2 | 10.9 | 10.9 | 10.7 | 10.5 | 10.9 | 10.8 | 11.3 |
| **40** | 11.2 | 11.1 | 10.8 | 10.8 | 10.7 | 10.4 | 10.8 | 10.8 | 11.3 |
| **50** | 11.0 | 11.0 | 10.7 | 10.8 | 10.7 | 10.4 | 10.8 | 10.8 | 11.3 |
| **60** | 11.0 | 10.9 | 10.7 | 10.8 | 10.7 | 10.4 | 10.8 | 10.8 | 11.3 |
| **70** | 10.9 | 10.8 | 10.6 | 10.7 | 10.7 | 10.3 | 10.8 | 10.8 | 11.2 |
| **80** | 10.8 | 10.7 | 10.6 | 10.7 | 10.7 | 10.3 | 10.8 | 10.8 | 11.2 |
| **90** | 10.7 | 10.6 | 10.5 | 10.6 | 10.7 | 10.3 | 10.7 | 10.8 | 11.2 |
| **100** | 10.6 | 10.5 | 10.5 | 10.6 | 10.7 | 10.3 | 10.7 | 10.8 | 11.2 |
| **110** | 10.5 | 10.4 | 10.4 | 10.6 | 10.7 | 10.2 | 10.7 | 10.8 | 11.2 |
| **120** | 10.4 | 10.3 | 10.4 | 10.6 | 10.7 | 10.2 | 10.7 | 10.8 | 11.2 |
| **130** | 10.4 | 10.2 | 10.3 | 10.5 | 10.7 | 10.2 | 10.6 | 10.7 | 11.2 |
| **140** | 10.3 | 10.2 | 10.3 | 10.5 | 10.7 | 10.2 | 10.6 | 10.7 | 11.2 |
| **150** | 10.3 | 10.1 | 10.3 | 10.5 | 10.7 | 10.1 | 10.6 | 10.7 | 11.1 |
| **160** | 10.2 | 10.1 | 10.2 | 10.5 | 10.7 | 10.1 | 10.6 | 10.7 | 11.1 |
| **170** | 10.2 | 10.1 | 10.2 | 10.5 | 10.7 | 10.1 | 10.6 | 10.7 | 11.1 |
| **180** | 10.2 | 10.0 | 10.2 | 10.5 | 10.7 | 10.1 | 10.6 | 10.7 | 11.1 |
| **190** | 10.2 | 10.0 | 10.2 | 10.5 | 10.7 | 10.1 | 10.5 | 10.7 | 11.1 |
| **200** | 10.1 | 10.0 | 10.2 | 10.5 | 10.7 | 10.1 | 10.5 | 10.7 | 11.1 |
| **210** | 10.1 | 10.0 | 10.2 | 10.5 | 10.7 | 10.1 | 10.5 | 10.7 | 11.1 |
| **220** | 10.1 | 10.0 | 10.2 | 10.5 | 10.7 | 10.1 | 10.5 | 10.7 | 11.1 |
| **230** | 10.1 | 10.0 | 10.2 | 10.5 | 10.7 | 10.1 | 10.5 | 10.7 | 11.1 |
| **240** | 10.1 | 10.0 | 10.2 | 10.5 | 10.7 | 10.1 | 10.5 | 10.7 | 11.1 |

## Section S2 – Viscosity studies

**Table S2-1 -** Viscosity of pure [$C_2$mim][SCN] and GDIL/[$C_2$mim][SCN] solutions before UV irradiation, at different temperatures.

| | Viscosity (mPa.s) | | | | | | | | | |
|---|---|---|---|---|---|---|---|---|---|---|
| **T (ºC) \ Solution** | **[$C_2$mim][SCN]** | **IMC1** | **IMC2** | **IMC4** | **IMC6** | **IMC8** | **IMBENZ** | **PY** | **CH** | **ET3N** |
| **20** | 28.067 | 27.930 | 27.904 | 27.786 | 28.263 | 28.186 | 28.432 | 28.354 | 27.822 | 27.896 |
| **25** | 23.590 | 23.479 | 23.453 | 23.364 | 23.742 | 23.685 | 23.877 | 23.815 | 23.391 | 23.450 |
| **30** | 20.072 | 19.979 | 19.951 | 19.882 | 20.191 | 20.143 | 20.305 | 20.249 | 19.909 | 19.953 |
| **35** | 17.265 | 17.194 | 17.168 | 17.108 | 17.364 | 17.328 | 17.459 | 17.414 | 17.133 | 17.171 |
| **40** | 14.999 | 14.946 | 14.915 | 14.867 | 15.079 | 15.060 | 15.164 | 15.126 | 14.892 | 14.92 |
| **45** | 13.144 | 13.101 | 13.076 | 13.036 | 13.216 | 13.201 | 13.289 | 13.252 | 13.059 | 13.083 |
| **50** | 11.614 | 11.579 | 11.561 | 11.522 | 11.676 | 11.668 | 11.74 | 11.711 | 11.545 | 11.565 |

**Table S2-2 -** Viscosity of pure [$C_2$mim][SCN] and GDIL/[$C_2$mim][SCN] solutions after UV irradiation, at different temperatures.

| | Viscosity (mPa.s) | | | | | | | | | |
|---|---|---|---|---|---|---|---|---|---|---|
| **T (ºC) \ Solution** | **[$C_2$mim][SCN]** | **IMC1** | **IMC2** | **IMC4** | **IMC6** | **IMC8** | **IMBENZ** | **PY** | **CH** | **ET3N** |
| **20** | 28.067 | 27.630 | 27.960 | 28.216 | 28.180 | 28.084 | 28.363 | 28.091 | 28.224 | 28.331 |
| **25** | 23.590 | 23.250 | 23.516 | 23.708 | 23.685 | 23.579 | 23.834 | 23.607 | 23.708 | 23.806 |
| **30** | 20.072 | 19.791 | 20.012 | 20.169 | 20.151 | 20.062 | 20.281 | 20.075 | 20.166 | 20.254 |
| **35** | 17.265 | 17.034 | 17.222 | 17.350 | 17.335 | 17.267 | 17.448 | 17.266 | 17.347 | 17.423 |
| **40** | 14.999 | 14.807 | 14.968 | 15.073 | 15.063 | 15.006 | 15.163 | 15.001 | 15.071 | 15.139 |
| **45** | 13.144 | 12.986 | 13.125 | 13.212 | 13.206 | 13.158 | 13.294 | 13.150 | 13.212 | 13.272 |
| **50** | 11.614 | 11.480 | 11.601 | 11.675 | 11.672 | 11.631 | 11.750 | 11.620 | 11.676 | 11.728 |

**Table S2-3 -** Viscosity of pure [$C_2$mim][$NTf_2$] and GDIL/[$C_2$mim][$NTf_2$] solutions before UV irradiation, at different temperatures.

| | Viscosity (mPa.s) | | | | | | | | | |
|---|---|---|---|---|---|---|---|---|---|---|
| Solution / T (ºC) | [$C_2$mim][$NTf_2$] | IMC1 | IMC2 | IMC4 | IMC6 | IMC8 | IMBENZ | PY | CH | ET3N |
| 20 | 39.825 | 37.780 | 38.735 | 38.598 | 38.540 | 37.967 | 36.590 | 35.747 | 35.770 | 36.447 |
| 25 | 33.153 | 31.561 | 32.288 | 32.159 | 32.119 | 31.681 | 30.568 | 29.914 | 29.897 | 30.448 |
| 30 | 27.948 | 26.672 | 27.254 | 27.149 | 27.110 | 26.766 | 25.856 | 25.343 | 25.309 | 25.768 |
| 35 | 23.833 | 22.803 | 23.271 | 23.184 | 23.153 | 22.875 | 22.116 | 21.693 | 21.658 | 22.042 |
| 40 | 20.540 | 19.688 | 20.078 | 20.004 | 19.975 | 19.749 | 19.107 | 18.751 | 18.721 | 19.044 |
| 45 | 17.873 | 17.159 | 17.484 | 17.421 | 17.395 | 17.206 | 16.655 | 16.360 | 16.331 | 16.608 |
| 50 | 15.678 | 15.075 | 15.352 | 15.299 | 15.276 | 15.118 | 14.633 | 14.389 | 14.362 | 14.602 |

**Table S2-4 -** Viscosity of pure [$C_2$mim][$NTf_2$] and GDIL/[$C_2$mim][$NTf_2$] solutions after UV irradiation, at different temperatures.

| | Viscosity (mPa.s) | | | | | | | | | |
|---|---|---|---|---|---|---|---|---|---|---|
| Solution / T (ºC) | [$C_2$mim][$NTf_2$] | IMC1 | IMC2 | IMC4 | IMC6 | IMC8 | IMBENZ | PY | CH | ET3N |
| 20 | 39.825 | 38.308 | 38.802 | 35.843 | 37.278 | 39.379 | 37.883 | 37.636 | 37.863 | 38.710 |
| 25 | 33.153 | 31.949 | 32.317 | 29.994 | 31.110 | 32.797 | 31.717 | 31.403 | 31.602 | 32.335 |
| 30 | 27.948 | 26.969 | 27.250 | 25.380 | 26.294 | 27.648 | 26.779 | 26.534 | 26.675 | 27.270 |
| 35 | 23.833 | 23.024 | 23.259 | 21.716 | 22.480 | 23.578 | 22.839 | 22.683 | 22.795 | 23.277 |
| 40 | 20.540 | 19.861 | 20.057 | 18.763 | 19.410 | 20.323 | 19.720 | 19.584 | 19.654 | 20.050 |
| 45 | 17.873 | 17.286 | 17.451 | 16.367 | 16.914 | 17.678 | 17.228 | 17.058 | 17.114 | 17.445 |
| 50 | 15.678 | 15.168 | 15.313 | 14.393 | 14.864 | 15.504 | 15.148 | 14.991 | 15.062 | 15.304 |

## Section S3 – Density studies

**Table S3-1 -** Density of pure [$C_2$mim][SCN] and GDIL/[$C_2$mim][SCN] solutions before UV irradiation, at different temperatures.

| | Density ($g.cm^{-3}$) | | | | | | | | | |
|---|---|---|---|---|---|---|---|---|---|---|
| T (ºC) \ Solution | [$C_2$mim][SCN] | IMC1 | IMC2 | IMC4 | IMC6 | IMC8 | IMBENZ | PY | CH | ET3N |
| 20 | 1.1193 | 1.1196 | 1.1196 | 1.1195 | 1.1194 | 1.1195 | 1.1197 | 1.1197 | 1.1197 | 1.1196 |
| 25 | 1.1163 | 1.1166 | 1.1166 | 1.1165 | 1.1163 | 1.1165 | 1.1167 | 1.1166 | 1.1167 | 1.1166 |
| 30 | 1.1133 | 1.1135 | 1.1136 | 1.1135 | 1.1133 | 1.1135 | 1.1137 | 1.1136 | 1.1136 | 1.1136 |
| 35 | 1.1103 | 1.1105 | 1.1106 | 1.1104 | 1.11033 | 1.1105 | 1.1107 | 1.1106 | 1.1106 | 1.1106 |
| 40 | 1.1073 | 1.1076 | 1.1076 | 1.1075 | 1.1073 | 1.1075 | 1.1077 | 1.1076 | 1.1077 | 1.1076 |
| 45 | 1.1043 | 1.1046 | 1.1047 | 1.1045 | 1.1044 | 1.1045 | 1.1047 | 1.1047 | 1.1047 | 1.1046 |
| 50 | 1.1013 | 1.1016 | 1.1017 | 1.1015 | 1.1014 | 1.1015 | 1.1017 | 1.1017 | 1.1017 | 1.1016 |

**Table S3-2 -** Density of pure [$C_2$mim][SCN] and GDIL/[$C_2$mim][SCN] solutions after UV irradiation, at different temperatures.

| | Density ($g.cm^{-3}$) | | | | | | | | | |
|---|---|---|---|---|---|---|---|---|---|---|
| T (ºC) \ Solution | [$C_2$mim][SCN] | IMC1 | IMC2 | IMC4 | IMC6 | IMC8 | IMBENZ | PY | CH | ET3N |
| 20 | 1.1193 | 1.1194 | 1.1196 | 1.1196 | 1.1196 | 1.1195 | 1.1197 | 1.1196 | 1.1196 | 1.1195 |
| 25 | 1.1163 | 1.1164 | 1.1166 | 1.1166 | 1.1165 | 1.1165 | 1.1166 | 1.1165 | 1.1166 | 1.1165 |
| 30 | 1.1133 | 1.1134 | 1.1136 | 1.1136 | 1.1135 | 1.1135 | 1.1136 | 1.1135 | 1.1136 | 1.1135 |
| 35 | 1.1103 | 1.1104 | 1.1106 | 1.1106 | 1.1105 | 1.1105 | 1.1106 | 1.1105 | 1.1106 | 1.1105 |
| 40 | 1.1073 | 1.1074 | 1.1076 | 1.1076 | 1.1075 | 1.1075 | 1.1076 | 1.1075 | 1.1076 | 1.1075 |
| 45 | 1.1043 | 1.1044 | 1.1046 | 1.1046 | 1.1046 | 1.1045 | 1.1047 | 1.1045 | 1.1046 | 1.1045 |
| 50 | 1.1013 | 1.1015 | 1.1017 | 1.1017 | 1.1016 | 1.1015 | 1.1017 | 1.1016 | 1.1017 | 1.1015 |

**Table S3-3 -** Density of pure [$C_2$mim][$NTf_2$] and GDIL/[$C_2$mim][$NTf_2$] solutions before UV irradiation, at different temperatures.

| | **Density ($g.cm^{-3}$)** | | | | | | | | | |
|---|---|---|---|---|---|---|---|---|---|---|
| **T (ºC) \ Solution** | **[$C_2$mim] [$NTf_2$]** | **IMC1** | **IMC2** | **IMC4** | **IMC6** | **IMC8** | **IMBENZ** | **PY** | **CH** | **ET3N** |
| **20** | 1.5242 | 1.5212 | 1.5229 | 1.5239 | 1.5239 | 1.5239 | 1.5241 | 1.5241 | 1.5241 | 1.5240 |
| **25** | 1.5192 | 1.5162 | 1.5179 | 1.5189 | 1.5189 | 1.5189 | 1.5191 | 1.5191 | 1.5191 | 1.5190 |
| **30** | 1.5142 | 1.5112 | 1.5129 | 1.5139 | 1.5139 | 1.5139 | 1.5140 | 1.5141 | 1.5141 | 1.5140 |
| **35** | 1.5092 | 1.5062 | 1.5079 | 1.5089 | 1.5089 | 1.5089 | 1.5091 | 1.5091 | 1.5091 | 1.5090 |
| **40** | 1.5042 | 1.4963 | 1.5029 | 1.5039 | 1.5039 | 1.5039 | 1.5041 | 1.5041 | 1.5042 | 1.5041 |
| **45** | 1.4992 | 1.4963 | 1.4979 | 1.4989 | 1.4990 | 1.4989 | 1.4991 | 1.4991 | 1.4992 | 1.4991 |
| **50** | 1.4943 | 1.4920 | 1.4930 | 1.4940 | 1.4940 | 1.4940 | 1.4942 | 1.4942 | 1.4943 | 1.4941 |

**Table S3-4 -** Density of pure [$C_2$mim][$NTf_2$] and GDIL/[$C_2$mim][$NTf_2$] solutions after UV irradiation, at different temperatures.

| | **Density ($g.cm^{-3}$)** | | | | | | | | | |
|---|---|---|---|---|---|---|---|---|---|---|
| **T (ºC) \ Solution** | **[$C_2$mim] [$NTf_2$]** | **IMC1** | **IMC2** | **IMC4** | **IMC6** | **IMC8** | **IMBENZ** | **PY** | **CH** | **ET3N** |
| **20** | 1.5242 | 1.5213 | 1.5235 | 1.5237 | 1.5237 | 1.5238 | 1.5245 | 1.5239 | 1.5240 | 1.5240 |
| **25** | 1.5192 | 1.5163 | 1.5185 | 1.5187 | 1.5187 | 1.5188 | 1.5195 | 1.5189 | 1.5190 | 1.5190 |
| **30** | 1.5142 | 1.5113 | 1.5135 | 1.5137 | 1.5137 | 1.5138 | 1.5145 | 1.5139 | 1.5140 | 1.5139 |
| **35** | 1.5092 | 1.5063 | 1.5086 | 1.5087 | 1.5087 | 1.5088 | 1.5095 | 1.5089 | 1.5090 | 1.5090 |
| **40** | 1.5042 | 1.5014 | 1.5036 | 1.5037 | 1.5037 | 1.5038 | 1.5045 | 1.5039 | 1.5040 | 1.5040 |
| **45** | 1.4992 | 1.4964 | 1.4986 | 1.4987 | 1.4988 | 1.4989 | 1.4996 | 1.4990 | 1.4991 | 1.4990 |
| **50** | 1.4943 | 1.4914 | 1.4937 | 1.4938 | 1.4938 | 1.4939 | 1.4946 | 1.4940 | 1.4941 | 1.4941 |
